# Unraveling the Role of the Tumor Extracellular Matrix to Inform Nanoparticle Design for Nanomedicine

**DOI:** 10.1002/advs.202409898

**Published:** 2024-12-04

**Authors:** Marco Cassani, Soraia Fernandes, Stefania Pagliari, Francesca Cavalieri, Frank Caruso, Giancarlo Forte

**Affiliations:** ^1^ International Clinical Research Center St. Anne's University Hospital Brno 60200 Czech Republic; ^2^ Department of Chemical Engineering The University of Melbourne Parkville Victoria 3010 Australia; ^3^ School of Science RMIT University Melbourne Victoria 3000 Australia; ^4^ School of Cardiovascular and Metabolic Medicine & Sciences King's College London London WC2R 2LS UK; ^5^ Dipartimento di Scienze e Tecnologie Chimiche Universita di Roma “Tor Vergata” Via della Ricerca Scientifica 1 Rome 00133 Italy

**Keywords:** cancer therapy, ECM, mechanobiology, mechanotherapy, nanomedicine

## Abstract

The extracellular matrix (ECM)—and its mechanobiology—regulates key cellular functions that drive tumor growth and development. Accordingly, mechanotherapy is emerging as an effective approach to treat fibrotic diseases such as cancer. Through restoring the ECM to healthy‐like conditions, this treatment aims to improve tissue perfusion, facilitating the delivery of chemotherapies. In particular, the manipulation of ECM is gaining interest as a valuable strategy for developing innovative treatments based on nanoparticles (NPs). However, further progress is required; for instance, it is known that the presence of a dense ECM, which hampers the penetration of NPs, primarily impacts the efficacy of nanomedicines. Furthermore, most 2D in vitro studies fail to recapitulate the physiological deposition of matrix components. To address these issues, a comprehensive understanding of the interactions between the ECM and NPs is needed. This review focuses on the main features of the ECM and its complex interplay with NPs. Recent advances in mechanotherapy are discussed and insights are offered into how its combination with nanomedicine can help improve nanomaterials design and advance their clinical translation.

## Graphical abstract

The extracellular matrix (ECM) plays a key role in cancer development and progression and significantly influences the therapeutic efficacy of nanomedicine. A better understanding of the nature of tumor ECM and mechanobiology is expected to contribute to the development of improved nanomaterial design and drug delivery, advancing the translation of nanoparticle‐based therapies in cancer treatment.

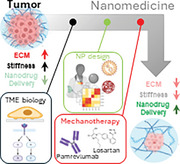

## 1 Extracellular Matrix Homeostasis

**Figure 1:**
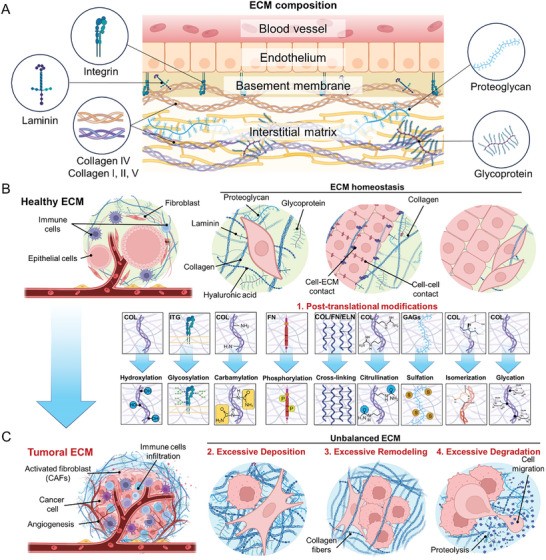
Structural and compositional desmoplastic ECM alterations occurring during cancer development. A) The ECM is generally divided into an interstitial matrix and a basement membrane, which is specific to different tissues and organs. The basement membrane, which is rich in laminins and collagen IV, provides structural and organizational stability to the upper cell layer. The interstitial matrix lies below the basement membrane and is rich in collagens, proteoglycans (such as hyaluronan), and glycoproteins (such as fibronectin).^[^ ^12^ ^]^ B) In a healthy microenvironment, epithelial cells interact with neighboring cells, including immune cells and resident fibroblasts, to maintain ECM homeostasis. C) However, during carcinogenesis, the ECM undergoes significant alterations, supporting a TME that sustains cancer development. These changes involve the activation of fibroblasts into cancer‐associated fibroblasts (CAFs) and the development of a fibrotic, stiff environment through: (1) posttranslational modifications of ECM components (collagen (COL), integrins (ITG), fibronectin (FN), elastin (ELN), glycosaminoglycans (GAGs); (2) excessive ECM deposition of collagen, laminin, hyaluronic acid, proteoglycans, and glycoproteins; (3) excessive ECM remodeling; and (4) excessive ECM degradation. These modifications support angiogenesis and the growth, survival, and invasion of tumor cells.

The extracellular matrix (ECM) is a noncellular component that constitutes the scaffolding structure of tissues and organs. In addition to providing structural support, the ECM delivers biochemical and mechanical cues to the embedded cells to initiate intracellular signaling pathways. The ECM is structured as a bioactive network of macromolecules assembled in a complex 3D architecture and presides over different physiological processes and cellular functions such as cell growth and motility.^[^ ^1^ ^]^ The composition and 3D structure of the ECM are tissue‐specific and show significant heterogeneity within the same tissue. The ECM undergoes continuous remodeling in a context‐specific fashion owing to traction forces and the enzymatic activity of cells with which it interacts.^[^ ^2^ ^]^ It is estimated that more than 300 core proteins and hundreds of associated proteins contribute to the so‐called matrisome; the core proteins are grouped into two main classes i.e., proteoglycans and fibrous proteins (collagens, elastins, fibronectins, and laminins).^[^ ^3^ ^]^ The ECM is formed by an interwoven interstitial matrix and a basement membrane (**Figure** **1**A). The interstitial matrix is mainly composed of different collagen types (such as collagens I and III), proteoglycans (such as hyaluronan), and glycoproteins (such as fibronectin and elastin) and forms the 3D network of the ECM connecting the cells within the stroma. The basement membrane is mainly composed of laminins, collagen IV, nidogens, and perlecan and controls cell organization and differentiation by direct interaction with cell surface receptors.^[^ ^3^, ^4^ ^]^ The importance of the ECM in the physiology of living tissues can be exemplified by the conditions and syndromes that are caused by mutations in ECM protein‐coding genes.^[^ ^5^ ^]^ Diseases such as Marfan syndrome, Ehlers‐Danlos syndrome (EDS), and osteogenesis imperfecta arise from genetic mutations that affect the synthesis or structure of ECM components, leading to weakened or improperly organized tissue structures.^[^ ^6^ ^]^

For instance, Marfan syndrome is an inherited disorder caused by autosomal dominant mutations in the fibrillin 1 protein.^[^ ^7^ ^]^ These defects reduce the amount of extracellular fibrillin‐rich microfibrils in the connective tissue, thus altering its organization and weakening its structural integrity. Additionally, in healthy conditions, these microfibrils act as a reservoir of transforming growth factor beta (TGF‐β), hence Marfan syndrome also results in dysregulated TGF‐β signaling.^[^ ^7^ ^]^ Marfan syndrome mainly affects the skeleton, eyes, and cardiovascular tissues but can also affect many other systems, as the connective tissues are widespread in the body.^[^ ^6^ ^]^

In a different example, EDS encompasses a group of disorders that primarily affect collagen types I, III, and V, which are essential for tensile strength in the ECM.^[^ ^8^ ^]^ Mutations in these collagen genes impair fibril formation and collagen cross‐linking, leading to structural weakness. Consequently, tissues with high collagen content, such as skin, joints, and blood vessels, become hyperflexible and fragile. Clinically, patients with EDS often present joint hypermobility, skin extensibility, and vascular complications due to the compromised structural integrity of the ECM.^[^ ^9^ ^]^

Another disease that affects collagen is osteogenesis imperfecta, which is mainly caused by mutations in collagen type I genes such as COL1A1 and COL1A2.^[^ ^10^ ^]^ Mutations in collagen type I—the main structural protein in bone—lead to improperly assembled collagen fibrils, resulting in reduced bone density, which renders the tissue prone to fractures.^[^ ^10^ ^]^

These conditions highlight how mutations in specific ECM components disrupt normal physiology, weakening tissue structure, altering cell signaling, and ultimately leading to the functional impairment of whole tissues or organs.

## 2 ECM in Cancer Biology and Therapy

As introduced in Section 1, in healthy conditions, the homeostasis of tissues relies on the precise regulation of ECM deposition, modification, degradation, and organization; these processes collectively define the biochemical and biophysical properties of the ECM (Figure 1B).^[^ ^11^ ^]^ During pathology, changes in the ECM can result from increased ECM deposition, posttranslational modification, proteolytic degradation, and force‐mediated physical remodeling (Figure 1C).^[^ ^11^ ^]^ In particular, ECM alteration has been described to occur in solid tumors, in a process termed desmoplasia. Specifically, desmoplasia refers to the fibrotic process that leads to the aberrant deposition of a dense and stiff ECM characterized by high cross‐linking and remodeling.^[^ ^12^ ^]^

Desmoplasia is known to occur in a wide range of solid cancers and is considered essential for tumor development and metastatic dissemination.^[^ ^3^ ^]^ Different cell types that compose the tumor microenvironment (TME) contribute to the formation and rearrangement of the ECM, including cancer cells and cancer‐associated fibroblasts (CAFs).^[^ ^3^, ^13^ ^]^ CAFs are highly represented in the tumorigenic stroma and are the main contributors to the production of the ECM.^[^ ^14^ ^]^ Through excessive deposition of ECM proteins, such as collagen and fibronectin, CAFs assist tumor progression and morphologically influence the polarity and conformation of epithelial cells, consequently impairing the physiology of the healthy epithelium.^[^ ^13^ ^]^ Consequently, tumor cells undergo epithelial‐to‐mesenchymal transition (EMT), switching toward a more aggressive phenotype. In addition, by activating matrix metalloproteinases (MMPs), continuous remodeling of the matrix paves the way for tumor cell migration and invasion.^[^ ^3^ ^]^

This evidence highlights the importance of mechanical cues in cancer development, making ECM deposition a primary target for adjuvant therapies aimed at halting tumor dissemination.

Numerous clinical trials are underway with the aim of assessing the efficacy of mechanotherapeutics on disease progression, as discussed further in Table 2. The term mechanotherapeutics refers to a drug or treatment targeting mechanotransduction pathways, with the aim of tackling tumor ECM deposition and remodeling.^[^ ^15^ ^]^ Several strategies have been proposed, which include the use of collagenases, metalloproteinases, hyaluronidases, and small molecules that can modulate ECM degradation and remodeling.^[^ ^16^ ^]^ More recently, a few strategies based on monoclonal antibody (mAB) delivery have been evaluated to target the desmoplastic transformation of the TME. For instance, the combination of trastuzumab (a mAB that targets human epidermal growth factor receptor 2 (HER2) in breast cancer) and hyaluronidase‐oysk (Herceptin Hylecta) has shown that the enzymatic digestion of the ECM, via hyaluronan degradation, facilitates the subcutaneous dispersion of the antibody.^[^ ^17^ ^]^ In a slightly different approach, simtuzumab, a mAB targeting lysyl oxidase‐like 2 (LOXL2), has been used to reduce fibrosis in several solid tumors, including liver, colorectal, and pancreatic cancer.^[^ ^18^ ^]^ LOXL2 catalyzes the cross‐linking of collagen and elastin, hence contributing to fibrotic ECM stabilization. The inhibition of LOXL2 has been shown to 1) suppress CAF activity, 2) reduce ECM deposition, 3) inhibit angiogenesis, and 4) prevent tumor cell invasion and metastasis.^[^ ^19^ ^]^ These results led to the investigation of simtuzumab in a phase II trial clinical study in patients with idiopathic pulmonary fibrosis and colorectal and pancreatic cancers. However, the study was discontinued as it did not support a clinical benefit.^[^ ^20^ ^]^ In a different example, pamrevlumab, a mAB against connective tissue growth factor (CTGF), is under phase III study as a neoadjuvant for chemotherapy in advanced unresectable pancreatic cancer (clinicaltrials.gov identifier: NCT03941093) and in metastatic pancreatic cancer (NCT04229004). CTGF, a secreted glycoprotein produced in different cell types including CAFs, can interact with several TME effectors such as TGF‐ β), vascular endothelial growth factor (VEGF), and integrins, playing a key role in fibrosis.^[^ ^21^ ^]^

Overcoming the physicochemical and biological barriers posed by the ECM has also been challenging for nanodrugs, formulations in which common chemotherapeutics are embedded or encapsulated into NPs.^[^ ^22^ ^]^ NPs enable the spatiotemporal distribution and control of drug delivery at the TME. Although some of the developed NPs have reached preclinical stages,^[^ ^23^ ^]^ efficient and effective NP delivery to tumors remains challenging because of 1) the limited and heterogeneous enhanced permeability and retention (EPR) effect among tumors, 2) ineffective targeting arising from protein corona formation on NPs, 3) rapid NP clearance by the mononuclear phagocytes system, 4) off‐target phenomenon with prominent nanodrug accumulation in the liver and other clearance organs, and 5) poor translational significance of results obtained in mouse models to humans.^[^ ^24^ ^]^

Only recently, the potential to integrate nanomedicine with mechanotherapy has emerged as an area of investigation.^[^ ^25^ ^]^ Although this combination of therapies holds promise to address solid tumor progression, it is important to elucidate the fundamental mechanistic processes that govern the interaction of NPs with ECM components. Understanding how these phenomena work in vitro and in vivo is essential for advancing cancer therapy.

## 3 ECM Mechanobiology

**Figure 2:**
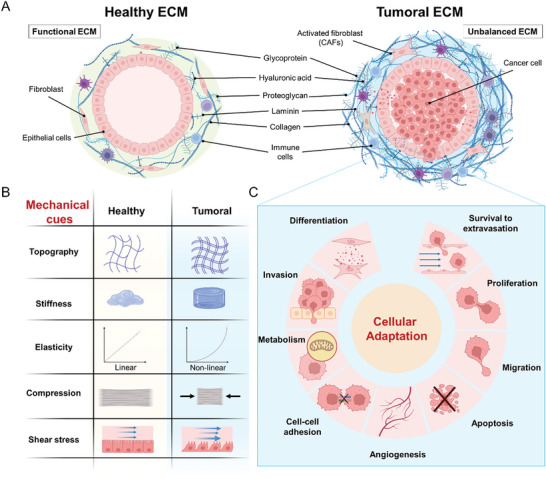
Mechanical cues in tumoral ECM support cancer development. A) In normal tissues, the epithelium is surrounded by a soft, compliant ECM that maintains tensional homeostasis (left panel). During carcinogenesis, proliferating epithelial cells exert outward compressive forces on the surrounding ECM, promoting CAF‐driven ECM remodeling, cross‐linking, and stiffening, which further contribute to immune infiltration (right panel). B) As a result of these escalating tensions, mechanical cues—such as topography, stiffness, elasticity, compression, and shear stress (stemming from interstitial pressure accumulation due to impaired lymphatic drainage and vessel compression)—become markedly distinctive of tumoral ECM. C) These mechanical cues contribute to further ECM and tumor tissue reorganization, stimulating angiogenesis and promoting cellular adaptation to the evolving microenvironment. This adaptation affects cell differentiation, proliferation, adhesion, migration, invasion, metabolism, and survival against programmed cell death and shear stress (e.g., during extravasation).

Cellular elasticity and cell‐generated forces are inherently interconnected.^[^ ^26^ ^]^ The intracellular and extracellular mechano‐responsive elements engage in constant, reciprocal interactions, linking mechanical signals with the cellular response.^[^ ^1^ ^]^ Together, these forces—referred to as mechanical cues—interact with intracellular signaling pathways to maintain mechanical balance (**Figure** **2**A), and regulate various cellular behaviors, including adhesion, spreading, receptor signaling, gene expression, and remodeling of the ECM.^[^ ^26^ ^]^

Mechanical cues include properties such as stiffness, elasticity, shear stress, compression, and topography of the surrounding ECM (Figure 2B; Section 3.1).^[^ ^27^ ^]^ The loss of mechanical homeostasis, along with the disruption of biochemical signaling, is a distinctive feature of the desmoplastic ECM.^[^ ^28^ ^]^ Posttranslational modifications of ECM components, their overexpression, excessive remodeling, and the deregulation of mechanosensing pathways all contribute to the imbalance in ECM homeostasis and the initiation of carcinogenic processes (see Figure 1).

Owing to the substantial alterations of the ECM occurring during carcinogenesis, tumors exhibit significant heterogeneity in their mechanical properties compared to normal and benign tissues. For instance, as cancer cells undergo transformation, they become increasingly compliant, with highly metastatic tumor cells being less stiff than normal cells.^[^ ^29^ ^]^ This variability in stiffness has been recognized as an important hallmark for cancer prognosis, and it has been shown to influence cancer cell behavior (Figure 2C).^[^ ^30^ ^]^

### 3.1 Definition of Mechanical Cues

**Mechanical stress** refers to the internal resistance of a material against an external force. It occurs when an object is subjected to forces that cause deformation. Stress is quantified as the amount of force applied per unit area of the material (N m^−2^, where N is newton and m is meter). There are three types of forces: **tension**, **compression**, and **shear**. Tension is caused by pulling forces that stretch the material; compression is caused by pushing forces that compress the material; shear stress is caused by forces that act parallel to the surface, leading to sliding between layers.

**Stiffness** indicates how much a material will deform (stretch or compress) in response to a given load. Stiffer materials deform to a lower extent under the same amount of stress compared with more flexible materials. It is measured in Pascal (Pa). Stiffness relates to elasticity, though stiffness may change as force increases.

**Elasticity** refers to the ability of a material to return to its original shape and size after the removal of an applied force. It describes how materials deform (stretch or compress) under stress and recover when the stress is released. **Linear elasticity** is directly proportional to the applied stress, meaning that linear elastic material will return to its original shape once the stress is removed if it is within the elastic limit. **Nonlinear elastic materials** can undergo substantial changes in shape without breaking when force is applied. Their response to stress does not follow a straight line. This means that as more force is applied, the relationship between the force and the resulting deformation becomes more complex and less predictable.

**Force** refers to an interaction that causes an object to change its motion, direction, or shape. It is typically defined as a push or pull acting upon an object. Force has both magnitude and direction, making it a vector quantity. It is measured in newton (N) and is represented by the symbol F.

**Figure 3:**
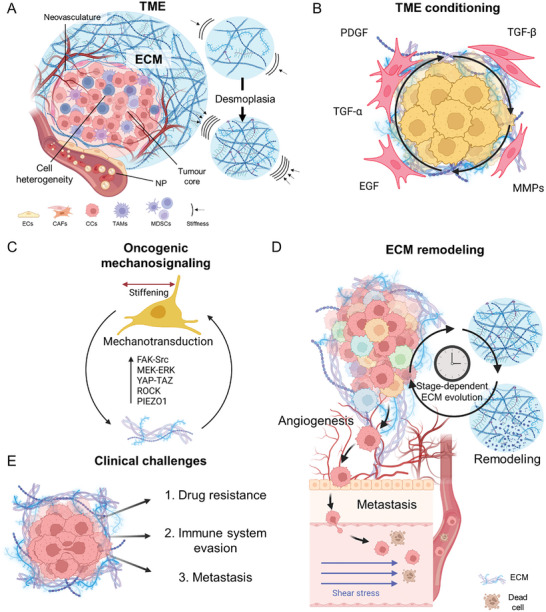
ECM composition, organization, and remodeling sustain the evolution of the TME and promote cancer progression and development. A) The TME is a complex system composed of various cellular and acellular components. The tumor ECM is significantly different from that in healthy tissues in composition and mechanical properties. An increased and abundant deposition of collagens and stiffening of the surrounding stroma is characteristic of pathology and, together with fiber reorganization, establish the hallmarks of desmoplasia.^[^ ^42^ ^]^ B) The irregular secretion of different factors (such as TGF‐β, PDGF, and EGF) and the increased activity of MMPs in the preneoplastic niche foster tumorigenicity and perpetuate the effects of desmoplasia. These occurrences facilitate the creation of a TME that supports the survival of cancer cells and the progression of the tumor. C) Mechanosensing pathways are commonly dysregulated in cancer cells, orchestrating the transcription of genes associated with cell proliferation, survival, and migration. A reciprocal loop forms between these pathways and ECM remodeling, mutually reinforcing one another and thereby promoting cancer progression. D) The presence of desmoplasia and the occurrence of ECM remodeling play a role in prompting cancer cells to adopt a more aggressive phenotype. This includes heightened migratory and invasive capabilities, which allow cancer cells to extravasate from the tumoral niche and enter the bloodstream, taking advantage of the newly formed vessels resulting from angiogenesis. During the process, some cells may evade clearance mechanisms and survive shear stress‐induced death, potentially leading to metastasis. E) The presence of tumor‐associated ECM presents several clinical challenges in cancer treatment. It acts as a physical barrier, limiting drug penetration and reducing the effectiveness of chemotherapy and immunotherapy, while releasing biochemical signals that promote immune evasion and foster drug resistance. It also enhances tumor cell migration and supports metastasis, which is responsible for most cancer‐related deaths. ECs, endothelial cells; CAFs, cancer‐associated fibroblasts; CCs, cancer cells.

The ECM is a highly dynamic system that is susceptible to alterations during disease progression. For example, post‐translational modifications and cross‐linking may significantly alter the properties of the ECM over time. Numerous cytokines and growth factors are tethered to the ECM proteins. Growth factors include hepatocyte growth factor (HGF), and members of the insulin‐like growth factor (IGF), fibroblast growth factor (FGF), and TGF‐β families. Continuous ECM remodeling determines the timely exposure and release of these cytokines and growth factors from storage, thereby contributing to tumor growth and development.^[^ ^31^ ^]^ Post‐translational modifications of specific ECM components, such as low molecular mass hyaluronan and collagens I, III, and V, along with the enzymatic activity of MMPs and LOXL2, are also involved in tumor development.^[^ ^3^ ^]^ Both the abundance and architecture of ECM components, as well as the interactions between different matrix elements and the presence of specific cell populations involved in fibrosis progression and matrix remodeling, are essential for developing a pro‐tumorigenic environment (**Figure** **3**A). In this regard, collagen XII has been demonstrated to regulate the organization of collagen I fibrils in a temporal dynamic fashion that promotes breast cancer cell invasion and metastasis.^[^ ^32^ ^]^ In a different example, netrin‐4, a basement membrane protein, has been shown to bind to laminin and modify its ternary structure, thus lowering ECM stiffness and reducing cancer cell invasion.^[^ ^4^ ^]^

Conversely, the excessive buildup and alteration of ECM proteins that lead to tumor stiffening are triggered by the abnormal expression or secretion of pro‐fibrotic proteins and factors within the TME (Figure 3B).^[^ ^33^ ^]^ Pro‐fibrotic elements present in the TME include TGF‐β, transforming growth factor α, platelet‐derived growth factor (PDGF), and epidermal growth factor (EGF). Moreover, tissue mechanosensing is intertwined with the ECM configuration.^[^ ^34^ ^]^ Various mechanosensing pathways are aberrantly activated upon ECM stiffening, perpetuating ECM remodeling and deposition (Figure 3C). This reciprocal interaction—known as “oncogenic mechanosignaling”—fosters tumor progression.^[^ ^34^ ^]^

The increased stiffness and altered chemical composition of the ECM both at cancer primary sites and in metastatic niches have also been shown to promote cell extravasation, a process critically involved in the formation of metastasis.^[^ ^35^ ^]^ Cell migration and intravasation are pivotal for disseminating cancer cells from a primary tumor to secondary sites within the body. The initial step of this process involves the intravasation of migrating cells into the bloodstream or lymphatic vessels (Figure 3D). Thus, migrating cells must breach the endothelial cell layer and withstand the damaging effects of shear stress present in the vasculature.^[^ ^36^ ^]^ Intravasation of tumor cells is often observed in regions characterized by low fluid shear, where angiogenesis‐induced abnormal and leaky tumor‐associated blood vessels are typically present.^[^ ^37^ ^]^

The presence of desmoplastic ECM poses several challenges to the clinical treatment of cancers (Figure 3E). Owing to its intricate 3D structure, desmoplastic ECM represents a major obstacle to drug diffusion toward solid tumors, thereby hindering the success of therapeutic interventions.^[^ ^3^ ^]^ Tumor drug resistance has been attributed to the low penetration and diffusion of chemotherapeutics through the abundant ECM.^[^ ^38^ ^]^

The ECM also hampers immune cell infiltration, preventing immune surveillance and tumor clearance, and thus facilitating tumor immune escape and progression.^[^ ^39^ ^]^ This is particularly undesirable when developing immunotherapies. A TME that is characterized by a stiff and compact ECM can prevent T cell infiltration and lead to T cell exhaustion, even under conditions where immunotherapy induces a cellular immune response.^[^ ^40^ ^]^

In this regard, it is important to highlight the significant role of the immune system in determining the course of the disease, as discussed elsewhere.^[^ ^41^ ^]^

**Figure 4:**
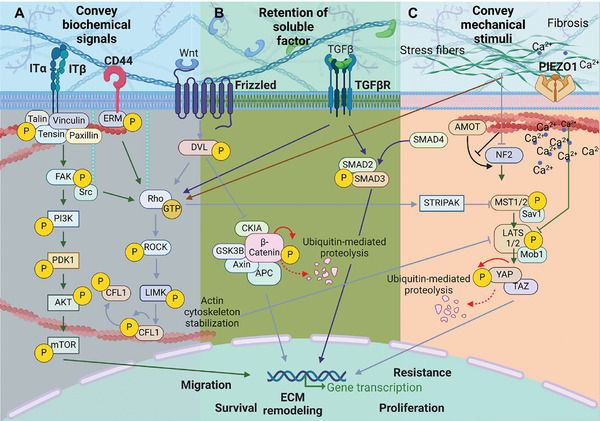
The aberrant tumorigenic ECM modulates several intracellular signaling pathways involved in tumor development and progression. A) The cell membrane is the main hub that conveys changes occurring at the ECM level into the cell; receptors on the cell membrane such as integrins, hyaluronan receptor (CD44), G protein‐coupled receptor (e.g., Frizzled receptor), and TGF‐β receptor play a major role in the mechanosignaling cascade. B) Importantly, the ECM directly interacts with these receptors and promotes the retention of secreted soluble factors (e.g., Wnt and TGF‐βB) at the TME, which can consequently be activated during disease development and fuel tumor progression by initiating a signaling cascade that can inhibit growth suppressors and pro‐apoptotic proteins (e.g., via FAK‐Src signaling), promote cell proliferation and differentiation (e.g., via Wnt/β‐catenin pathway), and enhance migration and invasiveness through EMT (e.g., via TGF‐β pathway).^[^ ^1^ ^]^ C) ECM stiffness also regulates the activity of Piezo1 ion channel, leading to the influx of Ca^2+^ ions and subsequent activation of downstream signaling pathways that modulate cell behavior. Mechanical cues also control the activity of angiomotin (AMOT) proteins and neurofibromin 2 (NF2, also known as Moesin‐Ezrin‐Radixin‐Like (MERLIN) tumor suppressor), which are negative regulators of yes‐associated protein (YAP). Following increased ECM stiffness, the assembly of actin results in the sequestration of AMOT, which prevents its interaction with NF2. This leads to the inhibition of the Hippo signaling pathway, resulting in the shuttling of YAP to the nucleus, where it promotes the transcription of genes encoding proteins that participate in focal adhesion assembly,^[^ ^44^ ^]^ immune evasion,^[^ ^45^ ^]^ resistance to therapy,^[^ ^46^ ^]^ and ECM remodeling.

During tumorigenesis, a positive feedback loop sustaining ECM deposition and remodeling is established between the mechanical cues from the TME and the intracellular biochemical signals.^[^ ^1^ ^]^ Cells can sense and respond to these stimuli through a process known as mechanotransduction, by which they convert mechanical signals into biochemical responses (**Figure** **4**). In these conditions, desmoplasia can direct the activation of several mechano‐sensitive signaling pathways that promote the proliferation, survival, migration, and invasion of cancer cells.^[^ ^1^ ^]^ Uncontrolled cell growth (characteristic of solid tumors), with cell spreading and sustained ECM deposition, results in increased stiffness at the TME, which consequently triggers Rho‐GTPase activity to maintain the integrity of the actin fibers composing the cytoskeleton. ECM stiffness also enhances angiogenesis and increases VEGF signaling in endothelial cells (ECs), which leads to the generation of neovasculature.^[^ ^43^ ^]^ Recent evidence has indicated that YAP translocation in the nucleus of breast cancer cells in contact with a stiff ECM reinforces cell–ECM interactions by promoting the expression of genes involved in focal adhesion assembly in a positive loop.^[^ ^44^ ^]^

Intracellular signaling pathways exhibit substantial overlap in the activation of their primary effectors downstream from the contact point between cells and ECM. Therefore, understanding the molecular mechanisms that guide TME remodeling, and consequently, devising effective therapeutic intervention, is challenged by stage‐of‐disease‐dependent activation of these pathways and the distinct roles of specific ECM components on the development of the pathology. At the cell membrane, integrins—the main point of contact with the ECM—ensure the mechanical connection between the cells and the surrounding microenvironment (Figure 4A). Integrins are a class of transmembrane heterodimeric proteins comprising 24 different subtypes in mammals, due to the combination of 18 α and 8 β subunits. Cell interaction with ECM structural proteins (such as fibronectin, collagen, vitronectin, and laminin) induces integrin clustering and recruitment of adaptor proteins. This process promotes the assembly of more mature multiprotein structures known as focal adhesions.^[^ ^47^ ^]^ Extracellular mechanical stimuli can dictate the organization of focal adhesions at the cell membrane and regulate the strength of binding between integrins and ECM components. This consequently promotes the activity of focal adhesion kinase and steroid receptor coactivator (FAK‐Src) signaling and the downstream activation of the Rho‐family GTPases ras homolog family member A (RhoA), ras‐related C3 botulinum toxin substrate (Rac), and cell division control protein 42 homolog (Cdc42), which control actin assembly in stress fibers.^[^ ^48^ ^]^ Healthy tissues generally display a softer ECM, attenuated FAK‐Src axes signaling, and enhanced activity of the Hippo pathway large tumor suppressor 1/2 kinase (LATS1/2). These conditions, together with the reduced GTPase Rho activity, promote the degradation of Yes‐associated protein/transcriptional coactivator with PDZ‐binding motif (YAP/TAZ) complex and prevent the shuttling of the complex into the nucleus. The presence of YAP/TAZ in the cytoplasm inhibits the formation and activation of specific co‐transcriptional complexes, and thus their co‐transcriptional activity.^[^ ^49^ ^]^ In contrast, ECM stiffening contributes to the activation of the mechanosensing YAP/TAZ axis.^[^ ^49c^ ^]^ Together with transcriptional enhanced associate domain (TEAD) transcription factors, YAP/TAZ leads to the expression of genes encoding for ECM‐related proteins, such as CTGF and CYR61, thus contributing to cell mechanics and cytoskeleton rearrangement.^[^ ^44^, ^50^ ^]^

Mechanical cues from the tumoral ECM can also promote actin assembly, enhancing cell motility and invasiveness, thus contributing to cancer progression via mechanically regulated Piezo channels ion‐channel receptors (Figure 4C).^[^ ^51^ ^]^ Through the mechanical forces exerted on the cell membrane, Piezo1 is directly regulated by ECM stiffness owing to its ability to convert mechanical cues of the extracellular environment into biochemical signals.^[^ ^52^ ^]^ When active, Piezo1 opens up, thus allowing the intracellular influx of calcium cations (Ca^2+^) that trigger several cancer signaling pathways including Akt/mechanistic targeting of rapamycin (mTOR) and YAP.^[^ ^53^ ^]^ Mechanical stimuli also influence the activity of AMOT and NF2 proteins, which act as negative regulators of YAP by preventing its shuttling into the nucleus and therefore inhibiting its transcriptional activity.^[^ ^54^ ^]^ Following an increase in cytoskeleton tension, AMOT is sequestered by forming actin bundles, and its binding to NF2 and YAP is disrupted. This leads to the inhibition of the Hippo pathway and the nuclear translocation of YAP.^[^ ^54^ ^]^

The aberrant TME remodeling occurring at tumor sites also influences TGF‐β and Wnt/β‐catenin‐mediated signaling pathways, favoring the extracellular retention of soluble factors TGF‐β and Wnt (Figure 4B).^[^ ^42^, ^55^ ^]^ Upon activation, these pathways serve a dual role. On one hand, they promote the nuclear shuttling of transcription factors mothers against decapentaplegic homolog (SMAD)2/3/4 and β‐catenin, which transcriptionally regulate the expression of genes involved in migration and invasiveness. On the other hand, they establish a positive feedback loop that enhances cell mechanics under mechanical stress conditions by activating RhoA‐GTPase.^[^ ^42^ ^]^

**Figure 5:**
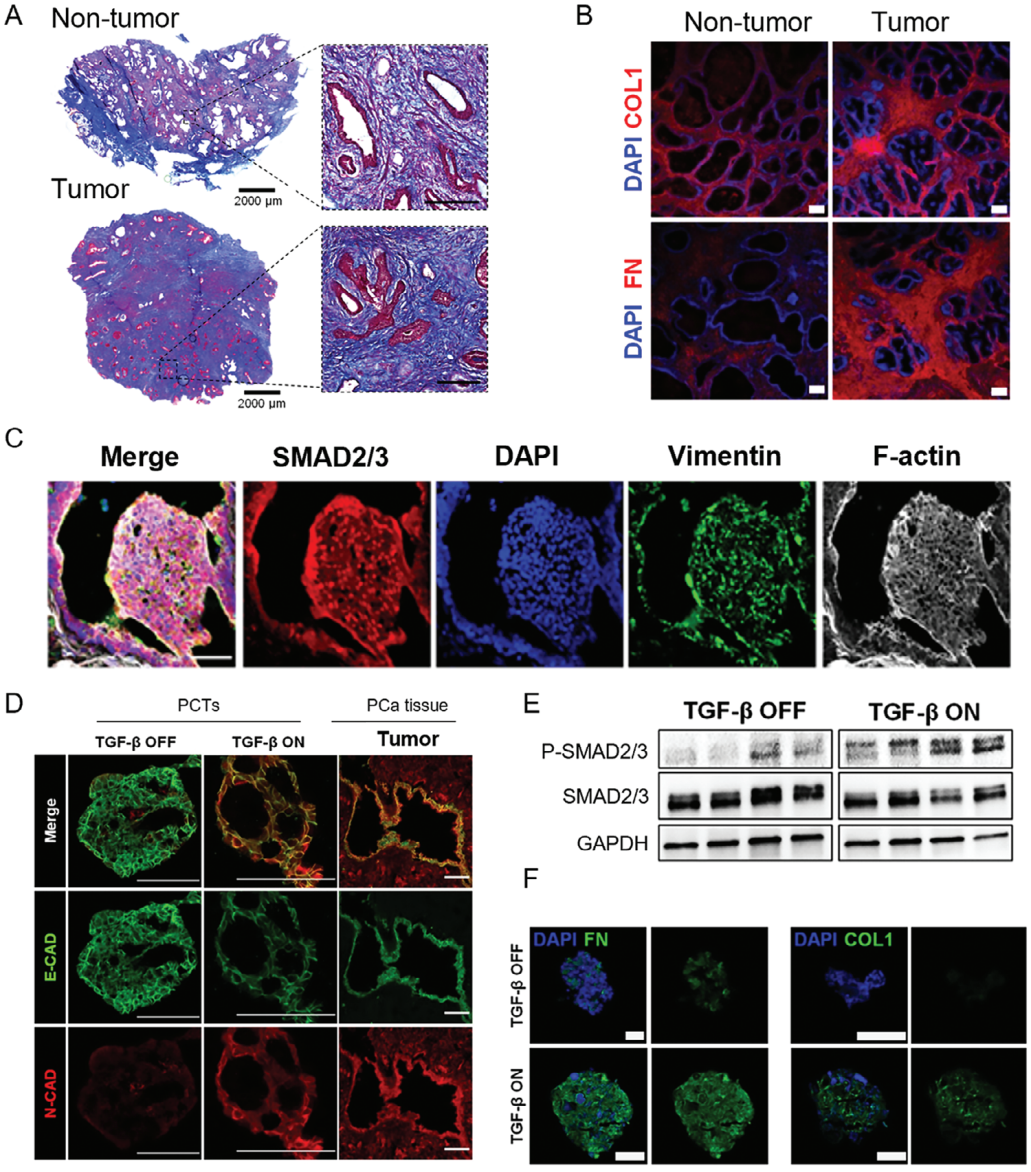
TGF‐β promotes prostate cancer (PCa) development by inducing desmoplasia, EMT, cancer cell invasion, and migration. A) Representative images of Masson's trichrome staining of tumor and adjacent non‐tumor prostate tissues. Collagen is stained in blue. Scale bars: 2000 and 200 µm (magnified area). B) Representative confocal microscopy images of the indicated ECM markers, such as collagen I (COL1) and fibronectin (FN), in tumor and adjacent non‐tumor prostate tissues. Scale bars: 200 µm. C) Representative confocal images of tumor tissues stained for 4′,6‐diamidino‐2‐phenylindole (DAPI) (blue), SMAD 2/3 (red), vimentin (green), and f‐actin (white). Scale bar in all images: 50 µm. D) Representative confocal images of the PCTs showing the expression levels of epithelial‐cadherin (E‐CAD, green) and N‐cadherin (N‐CAD, red)—markers of benign epithelium and EMT, respectively—in the presence or absence of TGF‐β pathway activation. The tumoroids were fixed with 4% paraformaldehyde and then embedded in optical cutting temperature compound for slicing, prior to immunofluorescence staining. Scale bars: 50 µm. E) Protein quantification by western blot of SMAD2/3 and phospho‐SMAD2 (p‐SMAD2), which is phosphorylated and activated downstream of the TGF‐β pathway. F) Representative confocal images depicting the expression of collagen I and fibronectin in PCTs in the presence or absence of TGF‐β pathway activation. Scale bars: 50 µm.^[^ ^58^ ^]^ Reproduced with permission.^[^ ^58^ ^]^ Copyright 2023, Elsevier B.V.

TGF‐β has been involved in the resistance of various anticancer treatments and in the regulation of the immune response by inhibiting tumor suppression and conferring resistance to checkpoint blockade therapies.^[^ ^56^ ^]^ Nevertheless, TGF‐β can display both pro‐ and anti‐tumorigenic functions depending on the stage of disease and type of cancer, thus affecting the outcome of different therapies that target this signaling pathway.^[^ ^57^ ^]^ In this context, we have recently demonstrated that TGF‐β signaling and tumor desmoplasia collaborate to promote cancer cell EMT and tumor progression in prostate cancer.^[^ ^58^ ^]^ By thoroughly examining a cohort of 80 patients, diagnosed with prostate cancer at different stages and undergoing radical prostatectomy, desmoplasia was identified as a hallmark of this type of cancer. Consistent ECM changes, including excessive deposition of collagen I and fibronectin, as well as alignment of collagen fibers were identified using various microscopy techniques. The role of TGF‐β in desmoplasia was confirmed by the presence of nuclear TGF‐β effector SMAD2/3 in prostate cancer tissues. Moreover, by modulating TGF‐β signaling pathway in 3D prostate cancer tumoroids (PCTs), established from patient samples, we revealed an interplay between TGF‐β expression and ECM accumulation and remodeling in primary prostate cancer. Through performing multi‐omics studies of the PCTs and a range of functional assays, we also demonstrated the relationship between TGF‐β ‐driven desmoplasia, EMT, and the migratory phenotype occurring in prostate cancer cells (**Figure** **5**).^[^ ^58^ ^]^

## 4 NP–ECM Interactions

Delivering NPs to solid tumors remains a significant challenge in achieving the successful clinical translation of nanomedicine, with estimates suggesting that only 0.7% of intravenously administered NPs reach the tumor.^[^ ^59^ ^]^ The clearance of NPs through renal and hepatic pathways, where the mononuclear phagocyte system (MPS) cells such as Kupffer cells can phagocytose a wide variety of particles, accounts for most of their removal from the body.^[^ ^60^ ^]^ In addition, the effects of the ECM structure and features, as well as properties inextricably linked to the TME (such as shear stress, hydrostatic pressure, and tension), on the penetration of nanomedicines into tumors pose a substantial challenge to cancer therapy.^[^ ^61^ ^]^

Aberrant ECM deposition impacts the delivery of NPs by trapping them in the extracellular space and hindering their diffusion toward the tumor.^[^ ^62^ ^]^ In addition, ECM composition and organization influence the mechanical state of the cells and their differentiation,^[^ ^63^ ^]^ which may impact their interaction with NPs. The correlation between cell mechanics, cell differentiation, and the endocytic pathway has been recently shown;^[^ ^64^ ^]^ the endocytic pathway depends on the activity of β‐catenin, RhoA, and ezrin–radixin‐moesin (ERM) proteins that determines an increase in membrane tension and a decrease in endocytosis. In addition, besides cancer cells, several cell types—such as CAFs and tumor‐associated macrophages (TAMs)—reside in the TME, contribute to tumor development, impact ECM remodeling, and determine the fate of nanomedicines by preventing NPs from reaching the desired therapeutic site.^[^ ^61^ ^]^ Therefore, understanding how ECM pathological remodeling drives disease development may help in designing more effective nanodrugs and improving the efficiency of cancer therapies.

**Figure 6:**
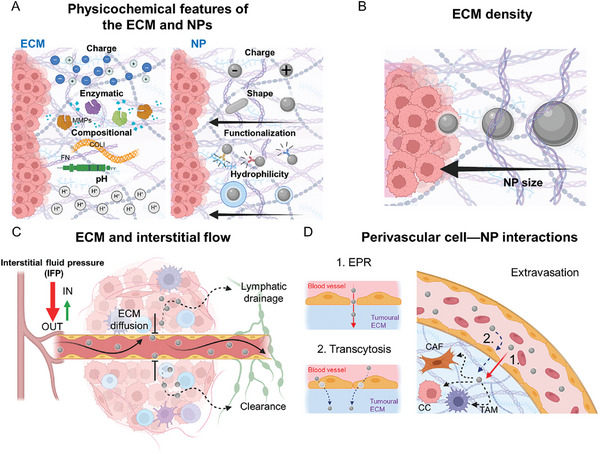
The different features of the ECM contribute to restricting the accumulation of NPs at the tumor site, thereby reducing the effectiveness of nanomedicines. A) The biochemical features of the ECM affect its interaction with NPs, which consequently influences the optimal physicochemical properties of the NPs—i.e., surface charge, shape, hydrophilicity, and surface functionalization. Considering the physicochemical properties of both the ECM and NPs, it is essential to evaluate the outcome of NP–ECM interactions. B) Concurrently, ECM density impairs the diffusion of NPs via steric hindrance. Consequently, smaller NPs (<50 nm) are better able to penetrate the ECM than larger NPs. C) Likewise, IFP limits the diffusion of drugs and NPs at the TME. Abnormal angiogenesis, increased matrix deposition, and fibrosis can alter the hydration of the stroma surrounding the tumor, creating an interstitial fluidic gradient that hinders the extravasation of small molecules or favors their clearance via lymphatic vessels.^[^ ^65^ ^]^ D) Two main mechanisms drive the accumulation of NPs in tumors: 1) EPR effect and 2) transcytosis. EPR relies on the leakiness of the fast‐growing vessels typical of the angiogenesis of tumors. Transcytosis involves the active internalization of the NPs by ECs of the blood vessels at the apical level and their subsequent exocytosis at the basal level. Although EPR has been demonstrated to be heterogeneous and inconsistent between animal and human tumors, and even among different human cancers, to date, transcytosis has only been observed in animal models. Once extravasated, NPs face interactions with and possible clearance by different cells residing at the TME, mainly by fibroblasts (CAFs) and macrophages (TAMs). Thus, targeting these cells may enhance the accumulation of NPs and drugs at the TME.

In the next section, we present the key factors that regulate NP–ECM interactions—as well as the interactions with the cellular components such as CAFs and TAMs that contribute to ECM physiology—and determine the fate of nanodrugs in the complex environment of a tumor (**Figure** **6**). We also discuss different strategies that have been employed in NP design to overcome detrimental interactions with the tumoral ECM (**Table** **1**).

**Table 1:** Overview of insights from selected studies on NP–ECM interactions.

Interaction^a)^	Key aspect	NP system	Mechanism	Ref.
**Physico‐chemical**	**Promote the optimal interaction of NPs with the tumoral ECM**	Gold NPs	High hydrophilicity and a negative surface charge facilitated NP diffusion across the ECM	[69b]
			ECM components, such as hyaluronan, can bind to the NPs and compete for the surface ligands	[67a]
		Pegylated‐quantum dots	Negatively charged ECM components, such as chondroitin sulfate, can hamper the diffusion of positive NPs in the ECM	[67b]
		Liposomes	The osmotic pressure generated by proteoglycans induces stresses that instigate the alteration of the structure of the lipid‐based particles	[68]
		Disk‐shaped NPs	Negatively charged, disk‐shaped NPs with a low aspect ratio penetrate the ECM more efficiently than spherical NPs or NPs with a high aspect ratio	[69, 70]
		Collagenase‐conjugated micellar NPs	The enzymatic activity of collagenase bound to the NPs increased the accumulation of the NPs in tumor	[71b]
		pH‐sensitive coating and linkers	pH‐Sensitive materials trigger the release of drugs in the acidic TME	[72]
		MMP‐2‐sensitive coating	The increased proteolytic processes occurring at the TME are exploited to increase the selective delivery of nanodrugs	[73]
		Legumain‐sensitive peptide	The increased proteolytic processes occurring at the TME are exploited to increase the selective delivery of nanodrugs	[75]
**Density**	**Reduce ECM density and alter its organization**	Gold nanorods	Physical methods such as PTT and MHT mediated by gold and iron oxide NPs respectively impair fibrillar collagen structure and promote tumoral ECM degradation, thus promoting NP penetration in the TME	[77a]
		Mesoporous nanorods	Papain‐loaded NPs for enhanced tumoral ECM degradation and PTT combining enzymatic and physical methods	[79b]
		Iron oxide NPs	Physical methods such as PTT and MHT mediated by gold and iron oxide NPs respectively impair fibrillar collagen structure and promote tumoral ECM degradation, thus promoting NP penetration in the TME	[81]
		Semiconductor polymer nanoenzyme	Near‐infrared irradiation enhances the collagen‐degrading activity of the nanoenzyme and increases PTT efficacy	[82]
		FAK siRNA/PD‐L1 siRNA‐loaded LNPs	The inhibition of FAK reduces ECM deposition and the stiffness of cancer cells, thus promoting the penetration of the LNPs in tumoral ECM and their internalization in cancer cells, while inhibiting immune checkpoint.	[25a]
**IFP**	**Increase the perfusion of NPs and drugs in the TME**	Imatinib/ DOX‐loaded liposomes	Imatinib reduces IFP and promotes the delivery of drugs to cancer cells	[86]
		Quercetin/ docetaxel‐loaded lipid nanocarriers	Imatinib reduces IFP and promotes the delivery of drugs to cancer cells	[87]
		Anti‐miR‐210/KRAS siRNA/CXCR4 antagonist‐loaded polymeric NPs	The codelivery of anti‐miR‐210 (to inactivate stroma‐producing pancreatic stellate cells), siKRAS^G12D^ (to kill pancreatic cancer cells), and CXRC4 antagonist (to block cancer‐stroma interactions) modulates the desmoplastic TME and improves cytotoxic T cells infiltration at the tumor	[88a]
		ATRA/HSP47 siRNA‐loaded gold NPs	The inhibition of pancreatic stellate cells through the combined delivery of ATRA and siRNA reduces ECM deposition, IFP, and consequently the anticancer efficacy of chemotherapeutics	[89]
		PFD‐loaded liposomes, MMP‐2‐responsive	PFD reduces IFP and hampers ECM deposition, thus promoting the delivery of chemotherapeutics to the tumor	[90b]
		Telmisartan‐loaded gelatin NPs in combination with paclitaxel‐loaded platinum NPs	The angiotensin II antagonist telmisartan reduces collagen deposition in tumoral ECM and IFP, promoting NP penetration and increasing the anticancer efficacy of drugs	[91]
		Hydralazine‐loaded liposomes	The antihypertension vasodilator hydralazine reduces tumor stroma and promotes NP penetration in tumors	[92]
		Losartan‐loaded CDs in combination with DOX‐loaded CDs	The angiotensin receptor 1 inhibitor losartan mitigates ECM deposition and hypoxia in the TME, enhancing the penetration of CDs and the release of DOX at the tumor	[93a]
**Perivascular cells**	**Limit the interaction of NPs with undesired cells or ECM components**	Paclitaxel/ vactosertib‐loaded polymeric NPs functionalized with EDB‐targeting peptide	Functionalization with the EDB‐targeting peptide increases the accumulation of the NPs at the tumor, while the release of TGF‐β inhibitor vactosertib further promotes NP accumulation and drug release by inhibiting fibrosis	[102a]
	**Exploit the targeting of CAFs and TAMs to accumulate NPs and chemotherapeutics at the tumor stroma**	sTRAIL plasmid‐loaded lipid‐coated protamine complexes functionalized with anisamide‐targeting CAFs	sTRAIL is transfected in CAFs and the released peptide promotes the apoptosis of the adjacent tumoral cells	[105]
		Photosensitizer‐loaded apoferritin NPs functionalized with FAP‐targeting scFv	In combination with PDT and immunotherapy, the NPs stimulate anticancer immunity and elicit immune responses against CAFs	[106]
		Cisplatin prodrug‐loaded polymeric NPs	TAMs engulf the NPs and act as a depot for the slow release of the cisplatin drug to the adjacent cancer cells	[110]
	**Combining NP‐based treatments with immunotherapies to restrain the immune‐suppressive TME**	Hyaluronidase‐conjugated dextran NPs in combination with photosensitizer‐loaded liposomes	Pretreatment with dextran NPs leads to tumoral ECM degradation and enhances PDT efficacy and penetration of cytotoxic T lymphocytes in tumoral ECM in combination with immunotherapy	[113]
		TGF‐β receptor inhibitor/PD‐L1 siRNA‐loaded pH‐responsive nanoclusters	The pH‐responsive NPs, sensitive to the acidic TME, can effectively reduce the deposition of collagen and promote the infiltration of cytotoxic T cells	[114]

^a)^ **NP**: nanoparticle; **ECM**: extracellular matrix; **TME**: tumor microenvironment; **MMP‐2**:matrix metalloproteinase 2; **PTT**: photothermal therapy; **MHT**: magnetic hyperthermia; **FAK**: focal adhesion kinase; **PD‐L1**: programmed death‐ligand 1; **LNPs**: lipid nanoparticles; **DOX**: doxorubicin; **IFP**: interstitial fluid pressure; **PFD**: pirfenidone; **CDs**: carbon dots; **KRAS**: Kirsten rat sarcoma virus, an oncogene encoding the K‐Ras protein, which is a component of the RAS/MAPK signaling pathway; **CXCR4**: C‐X‐C motif chemokine receptor 4, G protein‐coupled receptors directly involved in a number of biological processes including organogenesis, hematopoeisis, and immune response; **ATRA**: all‐trans retinoic acid; **EDB**: extra domain B splice variant of fibronectin; **TGF‐β**: transforming growth factor β; **sTRAIL**: secretable tumor necrosis factor (TNF)‐related apoptosis‐inducing ligand; **CAFs**: cancer‐associated fibroblasts; **PDT**: photodynamic therapy; **scFV**: single‐chain fragment variable; TAMs: tumor‐associated macrophages.

### 4.1 Physicochemical Properties of the ECM and NPs

The penetration of nanodrugs and, therefore, their efficiency at the tumor core can be significantly impaired by interactions with the surrounding ECM. Leveraging the properties of NPs and features of the desmoplastic ECM to optimize their interaction can maximize NP retention at the target site and help avoid undesired pitfalls. Hydrogen bonding and steric, hydrodynamic, electrostatic, and hydrophobic interactions at the interface between NPs and ECM components strongly affect NP diffusion throughout the ECM (Figure 6A).^[^ ^66^ ^]^ For example, the presence of negatively charged hyaluronan and chondroitin sulfate in the ECM was shown to hamper the diffusion of NPs by displacing or directly interacting with the coating molecules on the NP surface.^[^ ^67^ ^]^ Furthermore, the osmotic pressure generated by proteoglycans and other components of the ECM can influence the stability of NPs, promoting their aggregation and potentially reducing drug delivery efficiency.^[^ ^68^ ^]^ Anionic and hydrophilic NPs diffuse deeper within the TME than positive and hydrophobic NPs.^[^ ^69^ ^]^ The aspect ratio of the particles can also affect their delivery. For instance, it was reported that negatively charged, disk‐shaped NPs with a low aspect ratio (height/diameter ratio) penetrated better the ECM and were internalized to a greater extent than spherical NPs or NPs with a similar shape but with a higher aspect ratio.^[^ ^69^, ^70^ ^]^ Even among small NPs, e.g. gold NPs with diameters ranging between 2 and 5 nm, high hydrophilicity and a negative surface charge were found to facilitate diffusion across the ECM.^[^ ^69b^ ^]^

**Figure 7:**
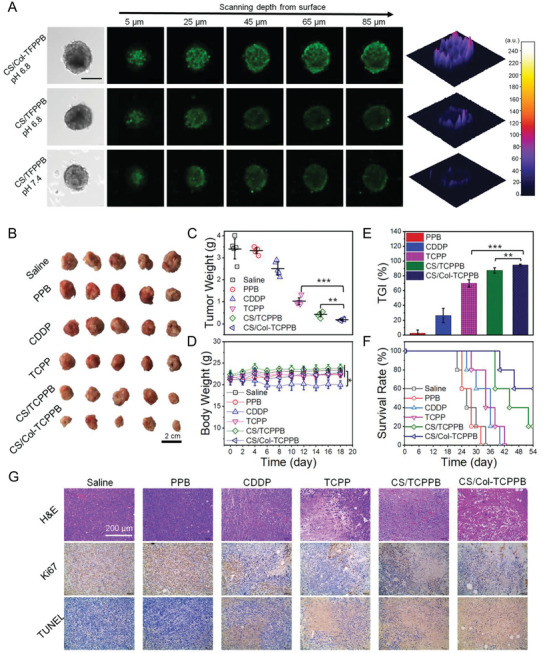
The use of biocompatible coatings to protect NPs and their cargo can increase the delivery efficacy at the TME of drugs and enzymes that are suitable for hampering the deposition and stiffening of ECM, thus increasing the anti‐tumoral efficacy of the nanoformulation. A) Representative confocal images showing the in vitro penetration of NPs in breast cancer spheroids; the NPs were coated with chondroitin sulfate, labeled with fluorescein isothiocyanate, and encapsulated collagenase enzyme (CS/col‐TFPPB); the CS/TFPPB formulation does not contain collagenase enzyme. Scale bar for all images: 200 µm. B) Efficiency of the nanoformulation was corroborated in vivo in 4T1 breast tumor‐bearing mice. Comparison of the images of tumors obtained from mice treated with control groups (saline, poly(ethylene glycol)‐block‐poly(β‐amino ester) (PPB), cis‐diamminedichloroplatinum (CDDP, cisplatinum), and succinic anhydride‐modified cisplatin‐conjugated poly(ε‐caprolactone)‐*block*‐poly(ethylene oxide)‐triphenylphosphonium (TCPP)) and the nanoformulations CS‐Col‐TCPPB and CS/TCPPB revealed a significant reduction in tumor volume for the group treated with CS‐Col‐TCPPB NPs. Scale bar: 2 cm. C) Comparison of the weight of the tumors excised from the different treated mice confirmed that CS‐Col‐TCPPB was more effective in halting tumor progression than that of other formulations. D) This efficacy was not associated with systemic toxicity, as only the free drug (CDDP) treatment was associated with a reduction in mouse body weight. E) Tumor growth inhibition (TGI) for the nanoformulations coated with chondroitin sulfate was significantly higher than that of the uncoated NPs, with the presence of collagenase further increasing TGI. F) The data were further confirmed by assessing the survival rate of the 4T1 tumor‐bearing mice treated with various regimens. G) Hematoxylin and eosin (H&E) staining, terminal deoxynucleotidyl transferase‐mediated dUTP nick‐end labeling (TUNEL), and immunohistochemical analyses (Ki67) were used to further evaluate the antitumor effect. In H&E staining, nuclei are stained blue and the cytoplasm is stained red. Ki67‐positive proliferating cells and TUNEL‐positive apoptotic cells are stained brown. Scale bar for all images: 200 µm.^[^ ^71b^ ^]^ Reproduced with permission.^[^ ^71b^ ^]^ Copyright 2020, WILEY‐VCH Verlag GmbH & Co. KGaA, Weinheim.

Several coatings have been used to decorate the surface of NPs to enhance nanodrug penetration and maximize their favorable interactions with the tumoral ECM. The presence of biocompatible and biodegradable moieties, such as chondroitin sulfate and erythrocyte membrane, resulted in increased circulation lifetime of particles and cargo protection from degradation or inactivation.^[^ ^71^ ^]^ Xu et al. developed a micellar NP composed of an assembly of maleimide‐terminated poly(ethylene glycol)‐*block*‐poly(β‐amino ester) and succinic anhydride‐modified cisplatin‐conjugated poly(ε‐caprolactone)‐*block*‐poly(ethylene oxide)‐triphenylphosphonium that could bind to thiolated collagenase through the maleimide groups of the *block*‐copolymer (**Figure** **7**).^[^ ^71b^ ^]^ The outer layer of this nanoformulation was coated with chondroitin sulfate, which formed a shell that protected collagenase from deactivation in blood circulation and overall increased the half‐life of the nanoformulation in vivo. The final size and surface charge of the obtained NPs were ≈100 nm and ≈−15 mV, respectively. The presence of collagenase in the NPs increased their accumulation in a model of breast cancer spheroids in vitro (Figure **7**A). Furthermore, the intravenous injection of the NPs in a murine breast tumor model showed that the presence of collagenase significantly inhibited tumor growth and increased the survival rate of the mice without causing systemic toxicity (Figure **7**B–G). Other approaches to target cancer cells have leveraged the metabolic properties of the TME. For example, pH‐sensitive coatings and linkers, such as ferritin heavy chains, alginate, and platinum prodrug‐conjugated poly(amidoamine), were used for the delivery of DOX, cisplatin, and collagenase; these pH‐sensitive materials triggered the release of drugs upon contact with the acidic environment of the tumor.^[^ ^72^ ^]^ In addition, the increased activity of proteases in the TME has been exploited to enhance the efficiency of nanodrug delivery. For example, an MMP‐2‐sensitive gelatin‐based coating was designed to exploit the activity of pro‐tumorigenic MMPs to increase the delivery and diffusion of DOX and NPs in the TME.^[^ ^73^ ^]^ In another study, polymeric micelles coated with a pro‐peptide sensitive to the cleavage of legumain (a lysosomal cysteine protease overexpressed in several cancer cells and macrophages at the TME)^[^ ^74^ ^]^ were used for the simultaneous release of the photosensitizer IR‐780 iodide and the kinase inhibitor sorafenib.^[^ ^75^ ^]^ This resulted in the effective penetration of the nanodrug into the tumor core and the reduction of cancer growth owing to the synergistic photothermal–chemotherapy treatment.^[^ ^75^ ^]^

### 4.2 ECM Density

**Figure 8:**
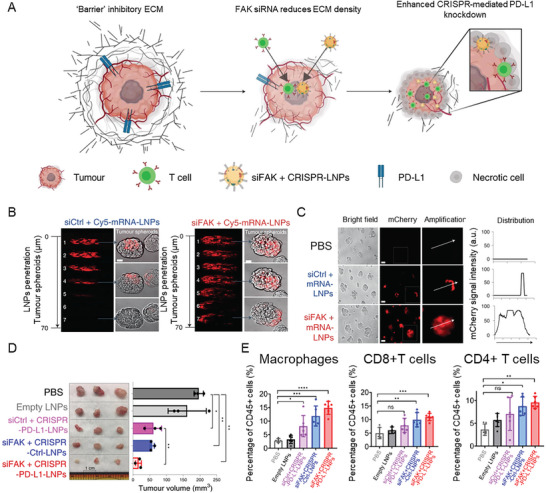
The combination of ECM‐targeting therapy and immunotherapy can enhance NP penetration into tumors and improve cancer therapy. A) Schematic of the mechanism of the delivery of LNPs encapsulating FAK siRNA, Cas9 mRNA, and targeted sgRNAs to the tumor. This approach aims to enhance NP penetration by targeting the mechanical properties of cancer cells while simultaneously improving immunotherapy by knocking down PD‐L1 via gene editing.^[^ ^25a^ ^]^ B) Representative images of the penetration of NPs into the IGROV1 tumor spheroids treated with LNPs carrying a scrambled siRNA (siCtrl + Cy5‐mRNA‐LNPs) or an siRNA against FAK (siFAK + Cy5‐mRNA‐LNPs) after 48 h incubation. Scale bar for all images: 50 µm. C) mCherry expression (left) and deep distribution (right) in tumor spheroids treated with phosphate‐buffered saline (PBS), siCtrl + Cy5‐mRNA‐LNPs, siFAK + Cy5‐mRNA‐LNPs, carrying mRNA for expressing the red fluorescent mCherry protein. Scale bars: 100 µm. D) Excised tumors (left) and tumor size (right) show the in vivo therapeutic efficacy of the LNPs carrying siFAK and CRISPR/Cas9 construct for PD‐L1 silencing in cancer cells (siFAK + CRISPR‐PD‐L1‐LNPs) and the other control groups in mice bearing ID8‐Luc xenograft tumors. Scale bar for all images: 1 cm. E) Flow cytometry analyses of macrophages, CD8+ T cells, and CD4+ T cells in the tumors from mice treated with PBS, cargo‐free (Empty) LNPs, siCtrl + CRISPR‐PD‐L1‐LNPs, siFAK + CRISPR‐Ctrl‐LNPs, or siFAK+CRISPR‐PD‐L1‐LNPs after 30‐day therapy. Reproduced with permission.^[^ ^25a^ ^]^ Copyright 2022, Nature.

The mesh size of collagen fibrils and interfibrillar proteoglycans and the content of glycosaminoglycans are responsible for the formation of a dense and highly interconnected protein network that can hamper the penetration of NPs.^[^ ^16^, ^76^ ^]^ It was shown that NPs larger than 50 nm accumulated at the periphery of the tumor vasculature, whereas smaller particles could penetrate deeper in the tumor (Figure 6B).^[^ ^77^ ^]^ Overall, an inverse correlation between NP size and the diffusion rate has been observed for many nanodrugs.^[^ ^78^ ^]^ Consequently, several approaches have been developed, focusing on the degradation of ECM using collagenases, metalloproteinases, hyaluronidases, and small anti‐fibrotic molecules such as α‐mangostin and pirfenidone (PFD).^[^ ^16b,c^ ^]^ The encapsulation of these molecules and enzymes inside NPs enables the preservation of the NP activity and their accumulation at the tumor site, where they can be released and can degrade the ECM components, thus facilitating NP penetration.^[^ ^16d^ ^]^ Similar strategies have been applied by encapsulating exogenous proteases, such as bromelain (extracted from pineapple) and papain (isolated from papaya), into inorganic NPs or polymeric micelles.^[^ ^79^ ^]^ As broad‐spectrum proteases, these exogenous enzymes are less specific than the endogenous counterparts but are highly stable and display optimal activity across a wide range of pH and temperature.^[^ ^79^ ^]^ Apart from enzymatic digestion, physical methods, such as photothermal therapy (PTT), photodynamic therapy (PDT), ultrasound therapy, and magnetic hyperthermia (MHT), can influence the architecture of the ECM and promote the penetration of NPs.^[^ ^80^ ^]^ For instance, PTT mediated by gold nanorods resulted in an altered fibrillar structure of collagen I and an increased penetration of the nanorods throughout the TME.^[^ ^77a^ ^]^ Similarly, MHT triggered by iron oxide NPs resulted in the reduction in the level of intact collagen fibers and degradation of the ECM structure.^[^ ^81^ ^]^ The application of PTT, PDT, and MHT using magnetic NPs can be combined with enzymatic treatment to improve the intratumoral accumulation of the NPs, thereby increasing the heat dose delivery.^[^ ^82^ ^]^ Alternatively, gene therapy against ECM deposition might be a promising strategy to target solid tumors. Recently, it was shown that the codelivery of lipid NPs (LNPs) carrying FAK siRNA and CRISPR‐PD‐L1 effectively reduced ECM deposition and stiffening of cancer cells, thus increasing transfection efficiency and resulting in knock‐down of immune checkpoint protein programmed death ligand 1 (PD‐L1) (**Figure** **8**A).^[^ ^25a^ ^]^ The LNPs were constructed using ionizable lipid 5A2‐SC8, cholesterol, 1,2‐dioleoyl‐*sn*‐glycero‐3‐phosphoethanolamine, 1,2‐dimyristoyl‐*rac*‐glycero‐3‐methoxy‐poly(ethylene glycol)‐2000 (DMG‐PEG2000), and DSPE‐PEG2000 at a molar ratio of 15:30:15:2:1. The obtained LNPs had a size of ∼120 nm and a surface charge of ∼10 mV. The specific composition selected for the synthesis of the LNPs facilitated endosomal escape. This enabled the concomitant release of siRNA targeting FAK and Cas9 mRNA with sgRNA targeting PD‐L1 (CRISPR gene editing), aiming to inhibit the expression of the immune checkpoint protein in the target cells. The inhibition of FAK reduced ECM deposition and stiffness and modulated the mechanical properties of cancer cells, resulting in an increased penetration of the LNPs in an in vitro spheroid model of IGROV1 cells (Figure 8B,C). In vivo studies on mice bearing ovarian tumors revealed that the reduction of ECM stiffness through gene silencing of FAK and the gene editing of PD‐L1, with the consequent suppression of the immune inhibition, effectively inhibited tumor growth and increased the infiltration of immune cells in the tumor tissue (Figure 8D,E).

Tackling desmoplasia can be achieved through a range of techniques, considering the properties of the nanomaterials and the presence of disease‐specific ECM components related to the activation of desmoplasia molecular pathways. The combination of these approaches and their administration at specific time points has the potential to effectively inhibit ECM deposition, enhance the penetration of nanodrugs, and reduce TME stiffness, thereby mitigating disease progression.

### 4.3 ECM and Interstitial Fluid Pressure

The interstitial flow within the TME differs significantly from that found in healthy tissues and is strongly influenced by ECM composition.^[^ ^65^ ^]^ For instance, high‐content proteoglycans, such as hyaluronan, are associated with increased interstitial flow, as these molecules can bind and trap water in the ECM.^[^ ^83^ ^]^ A disordered blood vessel network, a dense ECM, and rapid cell proliferation generate high interstitial fluid pressure (IFP), which is not conducive to drug delivery and NP accumulation into tumors (Figure 6C).^[^ ^84^ ^]^ Although small NPs can overcome the steric hindrance of the dense ECM, they still face rapid clearance from the tumor site due to the high IFP.^[^ ^78a^ ^]^ In this regard, a possible strategy to overcome IFP involves using polymers with temperature‐sensitive phase transition properties to synthesize polymeric NPs combined with PDT and PTT therapy.^[^ ^85^ ^]^ In one example, a thermoresponsive polymer, p(MEO_2_MA_160_‐*co*‐OEGMA_40_)‐*b*‐pSS_30_ was synthesized via atom transfer radical polymerization and used to form nanoaggregates loaded with the photosensitizer indocyanine green (ICG).^[^ ^85a^ ^]^ Following intratumoral injection of the nanoaggregates into 4T1 tumor‐bearing mice, light irradiation stimulated dendritic cells and cytotoxic T‐cells through the release of danger‐associated molecular patterns and induced immunogenic cell death. Noteworthy, due to the phase‐transition property of the polymer, the nanoaggregates remained in the tumor, thereby improving the PDT effect and minimizing phototoxicity to surrounding tissues.^[^ ^85a^ ^]^

**Figure 9:**
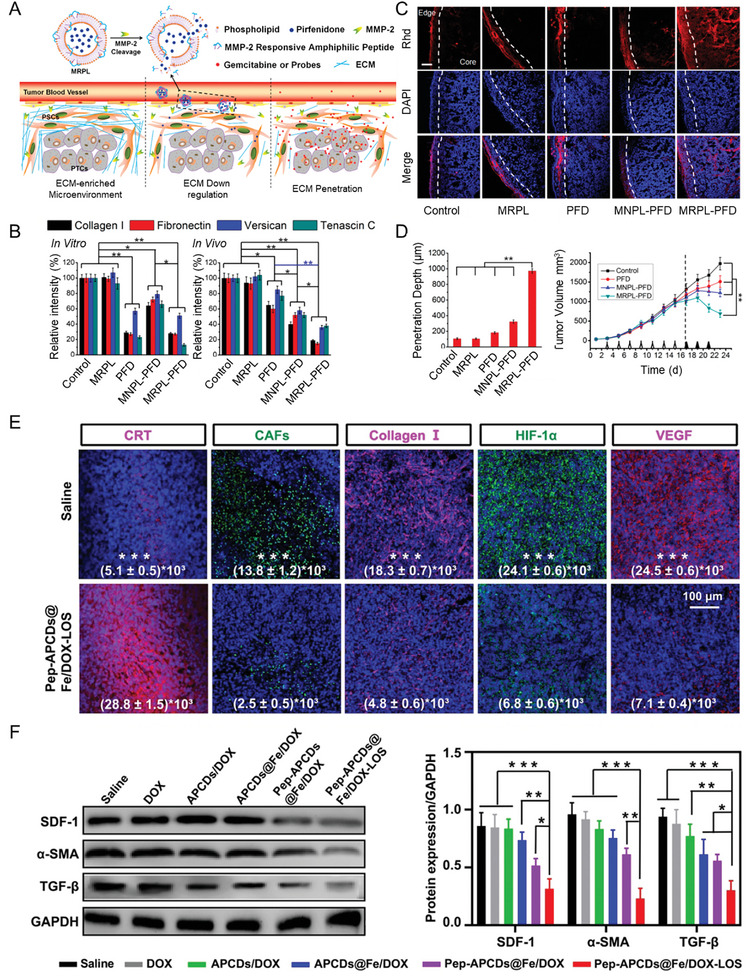
The use of drugs acting on the pathways involved in ECM deposition and organization can reduce IFP and improve the perfusion of nanomedicines and small molecules into tumors, thus improving their antitumor efficacy. A) Schematic of the delivery strategy of NPs loaded with PFD in the ECM‐enriched environment typical of pancreatic tumor. The NPs can achieve a tumor‐specific release of PFD in the tumor stroma via a process mediated by MMP‐2 activity. The subsequent release of PFD inhibits ECM deposition and increases the perfusion of small molecules in the tumor tissue. B) Expression levels of multiple ECM components (collagen I, fibronectin, versican, and tenascin C) from immunohistochemistry measurements derived from in vitro (left) and in vivo (right) treatments of pancreatic stellate cells and a murine pancreatic tumor model, respectively, with various formulations: MMP‐2‐responsive liposomes (MRPL) alone or encapsulating PFD (MRPL‐PFD) and MMP‐2‐nonresponsive liposomes (MNPL) alone or encapsulating PFD (MNPL‐PFD). C) Rhodamine (Rhd) penetration and distribution in a murine model of pancreatic tumor after 2 weeks of treatment with the different PFD formulations. Frozen tumor sections were stained with DAPI (blue) to label nuclei. Rhd is visible in red. Scale bar for all images: 100 µm. D) Quantification of the depth of Rhd penetration in tumors treated with the PFD formulations (left). Growth curves of pancreatic tumors in mice treated with the different PFD formulations and subsequently injected with gemcitabine (right).^[^ ^90b^ ^]^ E) Immunofluorescence analysis of calreticulin (CRT; red), CAFs (green), collagen I (red), hypoxia‐inducible factor‐1α (HIF‐1α) (green), and VEGF (red) at the endpoint after treatment with CD nanoassemblies carrying DOX and LOS (Pep‐APCDs@Fe/DOX‐LOS) compared to control saline treatment. Scale bar for all images: 100 µm. F) Western blot analysis showing the protein levels of SDF‐1, α‐SMA, and TGF‐β found in 4T1 tumor‐bearing mice after treatment with different nanoformulations (left) and the relative quantification (right).^[^ ^93a^ ^]^ (A–D) Reproduced with permission.^[^ ^90b^ ^]^ Copyright 2017, American Chemical Society. (E, F) Reproduced with permission.^[^ ^93a^ ^]^ Copyright 2020, Wiley‐VCH GmbH.

Other approaches have been focused on directly targeting IFP and the molecular effectors that contribute to it. Tyrosine kinase inhibitor imatinib (against platelet‐derived growth factor receptor β) and bevacizumab (a mAb against VEGF) have been encapsulated into NPs and used in different therapeutic strategies to reduce IFP. For instance, liposomes loaded with imatinib and DOX reduced IFP and promoted the delivery of DOX to cancer cells.^[^ ^86^ ^]^ In another study, imatinib was encapsulated into nanostructured lipid nanocarriers and delivered in combination with docetaxel and quercetin (with phosphatidylinositol 3‐kinase inhibitory activity), demonstrating interstitial flow reduction and improved antitumor efficacy.^[^ ^87^ ^]^ In addition, several other inhibitors have been used as anti‐fibrotic drugs to hamper ECM deposition and reduce IFP, including miRNA,^[^ ^88^ ^]^ siRNA,^[^ ^89^ ^]^ PFD,^[^ ^90^ ^]^ telmisartan,^[^ ^91^ ^]^ and hydralazine.^[^ ^92^ ^]^ For example, Ji et al. developed an MMP‐2‐responsive peptide hybrid liposome by co‐assembly of a tailor‐designed MMP‐2‐responsive amphiphilic peptide with a phospholipid (l‐α‐phosphatidylcholine). The liposomes were loaded with PFD for targeted delivery of the drug at the tumor site. The NPs were ≈65 nm in size and displayed a negative surface charge of ≈−22 mV (**Figure** **9**A).^[^ ^90b^ ^]^ The PFD‐loaded liposomes effectively reduced the deposition of key ECM components secreted by pancreatic stellate cells, such as collagen I, fibronectin, versican, and tenascin C, both in vitro and in vivo, in a murine model of pancreatic tumor model (Figure 9B). The long‐term treatment of the tumor in vivo with the protease‐responsive liposomes loaded with PFD increased the intratumoral accumulation of small molecules, such as rhodamine, and the therapeutic efficacy of gemcitabine drug (Figure 9C,D). In the context of drug delivery for reducing IFP and improving tumor perfusion, losartan (LOS) has been applied in several preclinical studies.^[^ ^93^ ^]^ LOS is an angiotensin receptor II antagonist that exerts its adjuvant activity by reducing fibrosis and IFP.^[^ ^94^ ^]^ For example, Hou et al. reported the preparation of nanoassemblies of carbon dots (CDs) functionalized with an FAP‐responsive peptide for the codelivery of DOX and LOS.^[^ ^93a^ ^]^ The amine‐rich CDs were cross‐linked by the carboxyl groups of the responsive peptide Asp‐Ala‐Thr‐Gly‐Pro‐Ala to produce mesoporous nanoassemblies. Subsequently, DOX molecules were loaded via π–π stacking interactions into the nanoassemblies, and Fe ions were chelated on the surface of CDs, while LOS was trapped in mesopores that were generated during NP formation. The final hydrodynamic diameter of the NPs was ≈106 nm and their surface charge was slightly positive (≈ +2 mV). In vivo treatment of 4T1 tumor‐bearing mice with the nanoassemblies carrying LOS resulted in a significant reduction of fibrosis and hypoxia, consequent to the decrease in deposition of collagen I, hypoxia‐inducible factor‐1a and VEGF (Figure 9E). As consistent with these findings, a reduction was observed in the protein levels of stromal cell‐derived factor‐1 (SDF‐1) and alpha‐smooth muscle actin (α‐SMA)—two canonical markers of fibroblasts involved in the activation of CAFs—and TGF‐β in the tumor (Figure 9F).

Recently, the treatment of advanced pancreatic ductal adenocarcinoma with a combination of LOS and FOLRINOX (therapeutic regimen composed of folinic acid, fluorouracil, irinotecan, and oxaliplatin) has demonstrated enhanced efficacy and downstaging of the disease.^[^ ^95^ ^]^ By inhibiting the angiotensin system, LOS reduces collagen and hyaluronan production by suppressing pro‐fibrotic signals such as TGF‐β1, thus improving the delivery of chemotherapeutics.^[^ ^96^ ^]^ In addition, TGF‐β, Wnt/β‐catenin, and Hedgehog signaling pathways have been successfully targeted to remodel the ECM structure, reducing its density and consequently the IFP.^[^ ^97^ ^]^

In this regard, a comprehensive understanding of the pathways that drive desmoplasia in different types of cancers and stages is expected to contribute to defining the timely application of a therapy targeting several molecular features of the disease.

### 4.4 Perivascular Cell Interactions

**Figure 10:**
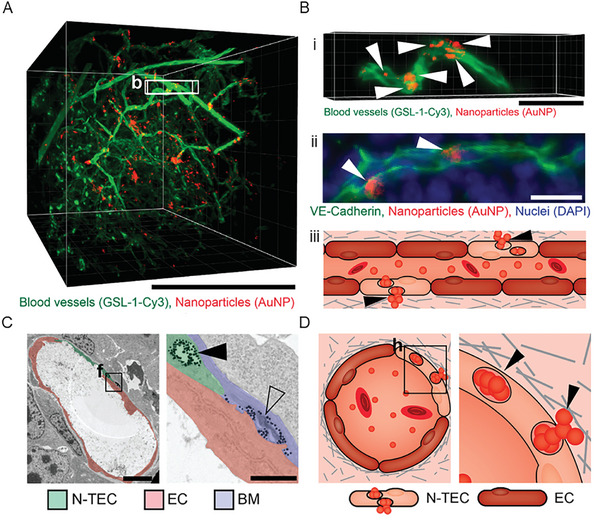
The accumulation of NPs at the tumor can be mediated by processes beyond the classical EPR effect, such as transcytosis by a specific subset of ECs present in the tumor vasculature. A) 3D microscopy image of murine tumor derived from 4T1 cells showing blood vessels in green and 50 nm gold NPs in red. Scale bar: 500 µm. B) Magnified region of (A) (b‐squared selection) showing an individual vessel with clusters of NPs, indicated by white arrows (i). 2D section of a representative blood vessel stained with vascular endothelial (VE)‐cadherin to highlight EC boundaries; clusters of NPs are highlighted with white arrows (ii). Schematic showing NP transport mediated by specific ECs; black arrows indicate clusters of NPs (iii). Scale bar: 50 µm. C) Transmission electron microscopy image of a representative tumor blood vessel (left) and a close‐up image of a region of the vessel where NPs accumulated (right). The solid black arrow shows NPs packaged in a vacuole of an endothelial cell (N‐TEC). The open arrow shows NPs in the extravascular space surrounded by the basement membrane. The N‐TEC is highlighted in green, cells without NP uptake are in red, and the basement membrane is shown in blue. Scale bars: 5 µm (left), 1 µm (right). D) Schematic of the mechanism of NP transcytosis by N‐TECs at the interface between blood vessels and the extracellular space. Clusters of NPs are indicated by black arrows.^[^ ^101^ ^]^ Reproduced with permission.^[^ ^101^ ^]^ Copyright 2021, American Chemical Society.

Endothelial cells (ECs) in the vessels are the first cellular barrier that NPs encounter during circulation in the bloodstream (Figure 6D). A common topic of discussion in nanomedicine is the presence of a leaky vasculature at tumor sites, which would favor the delivery of NPs by passive targeting through the EPR effect.^[^ ^98^ ^]^ However, EPR has been shown to be a heterogeneous phenomenon, with significant differences observed between murine models and human subjects.^[^ ^99^ ^]^ More recently, transcytosis has been proposed as an alternative mechanism describing NP extravasation from the bloodstream into the tumor site.^[^ ^100^ ^]^ For example, Kingston et al. demonstrated that the accumulation of 50 nm PEGylated (methoxy‐poly(ethylene glycol)‐thiol, 5 kDa) gold NPs in a murine breast tumor model was driven by specific tumor ECs (referred to as nanoparticle transport endothelial cells; N‐TECs), which allowed the transport of NPs from the lumen of blood vessels into the TME (**Figure** **10**).^[^ ^101^ ^]^ This mechanism is a size‐dependent process that occurs for NPs smaller than 100 nm. It has been shown that free drugs, such as cisplatin, tend to extravasate in a passive way and mainly through a leaky tumoral endothelium.^[^ ^99^, ^100^ ^]^ These findings may also explain the higher penetration rate generally observed for smaller NPs in the TME, as discussed above.

**Figure 11:**
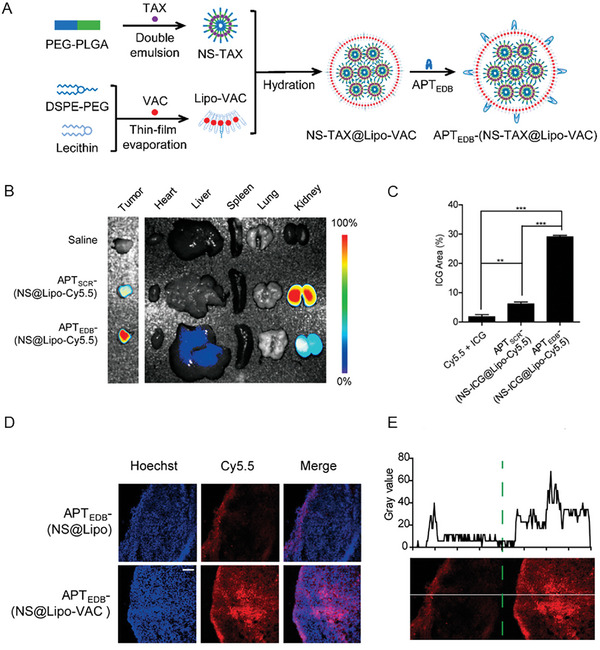
The modulation of the TME via the inhibition of pro‐fibrotic cells and the reduction of ECM deposition can increase the accumulation of nanodrugs at tumors. A) Schematic of the synthesis of NPs (specifically nanospheres (NS)) encapsulating vactosertib (VAC) and paclitaxel (TAX) (NS‐TAX@Lipo‐VAC) and functionalized with a peptide specific for the fibronectin EDB (APT_EDB_) (APT_EDB_‐NS‐TAX@Lipo‐VAC). PLGA, poly(lactic‐*co*‐glycolic acid). B) NPs functionalized with APT_EDB_ or a scrambled peptide (APT_SCR_) and labeled with Cy5.5 dye were injected intravenously into mice with orthotopic pancreatic cancer. Ex vivo Cy5.5 fluorescence images of major organs and tumors show the prevalent accumulation of the NPs functionalized with APT_EDB_ at the tumor. C) The results were further confirmed with photoacoustic imaging, by encapsulating ICG into the nanoformulation, which revealed the higher accumulation of the APT_EDB_‐functionalized NPs at the tumor compared to the NPs functionalized with APT_SCR_. D) By adding vactosertib to the nanoformulation, the NPs functionalized with APT_EDB_ showed higher accumulation at the tumor, and E) could penetrate deeper into the tumoral mass compared to the NPs without the drug.^[^ ^102a^ ^]^ Reproduced with permission.^[^ ^102a^ ^]^ Copyright 2021, American Chemical Society.

A strategy proposed to enhance the specificity of nanodrugs and prevent their nonspecific uptake by stromal cells involves targeting ECM proteins such as extra domain B (EDB) of fibronectin and tenascin C, which are abundant in the tumor ECM. Using this strategy, the accumulation of agents—such as the cytotoxic drug paclitaxel or antifibrotic and pro‐inflammatory molecules (e.g., α‐mangostin and TNF superfamily member 14, also known as LIGHT cytokine)—in the TME has been demonstrated to suppress fibrosis and tumor growth while stimulating the immune response.^[^ ^102^ ^]^ For example, Zhao et al. designed NPs that targeted the EDB of fibronectin, an isoform of the protein abundant in the TME of pancreatic ductal adenocarcinoma, and encapsulated paclitaxel and vactosertib, which is a TGF‐β1 inhibitor (**Figure** **11**A).^[^ ^102a^ ^]^ Specifically, a size‐switchable nanosystem was devised by incorporating paclitaxel within the hydrophobic layer of small PEG‐poly(lactic‐*co*‐glycolic acid) nanospheres, which were encapsulated into PEGylated liposomes carrying vactosertib within the hydrophobic core of the phospholipid bilayer. The PEGylated liposome consisted of lecithin, 1,2‐distearoyl‐*sn*‐glycero‐3‐phosphoethanolamine‐*N*‐[methoxy(polyethylene glycol)‐2000] (DSPE‐mPEG2000), 1,2‐distearoyl‐*sn*‐glycero‐3‐phosphoethanolamine‐*N*‐[maleimide(polyethylene glycol)‐2000] (DSPE‐PEG2000‐Mal), and cholesterol at molar ratio 10:2:1:4:1.7. The NPs were subsequently functionalized with an EDB‐targeting peptide. The obtained NPs displayed a hydrodynamic diameter of ≈224 nm and a surface charge of ≈−25 mV. The NPs functionalized with the EDB‐targeting peptide accumulated at the tumor to a higher extent than NPs functionalized with a scrambled peptide (Figure 11B,C). Furthermore, the addition of vactosertib to the NP formulation led to an increase in the tumor penetration of the NPs, which accumulated into the inner part of the tumor owing to the inhibitory effect of vactosertib on the pro‐fibrotic activity of pancreatic stellate cells (Figure 11D,E).

Cells other than ECs that reside in the perivascular region can also determine the fate of NPs upon extravasation. CAFs and TAMs are the most represented cells in the TME and actively contribute to ECM remodeling.^[^ ^103^ ^]^ For instance, CAFs were found to uptake most NPs in a study involving the delivery of lipid‐coated calcium phosphate NPs to tumor spheroids; the NPs were 18 and 50 nm in size and displayed a positive surface charge of ≈20 mV.^[^ ^104^ ^]^

Considering the different cellular components of the TME and their role in impairing nanodrug accumulation at the tumor core, NPs can be used to target CAFs or TAMs in combination with chemo‐ and/or immunotherapies to enhance the success of anticancer therapies.

CAFs have been proposed as eligible targets for NP delivery aimed at suppressing their pro‐fibrotic activity and possible normalization of ECM production and deposition. This strategy has been exploited to deliver a plasmid encoding the secretable tumor necrosis factor (TNF)‐related apoptosis‐inducing ligand (sTRAIL) via lipid‐coated protamine complexes composed of 1,2‐dioleoyl‐3‐trimethylammonium‐propane (DOTAP) and cholesterol at a molar ratio of 1:1, followed by the post‐insertion of 1,2‐distearoyl‐*sn*‐glycero‐3‐phosphoethanolamine‐*N*‐[methoxy(polyethyleneglycol‐2000) (DSPE‐PEG2000)] and DSPE‐PEG‐anisamide for CAF targeting purpose.^[^ ^105^ ^]^ The final particles had a diameter of ≈70 nm and a surface charge of ≈25 mV. The plasmid was effectively transfected into CAFs and the released sTRAIL induced apoptosis in adjacent tumor cells.^[^ ^105^ ^]^ In another study, a single‐chain fragment variable (scFv) against fibroblast‐activation protein (FAP) was used to functionalize apoferritin NPs that were loaded with a photosensitizer (ZnF_16_PC).^[^ ^106^ ^]^ In combination with PDT and anti‐programmed cell death protein 1 therapy, the NPs stimulated anticancer immunity and elicited immune responses against CAFs.^[^ ^106^ ^]^ Noteworthy, although CAFs are generally considered as tumor‐promoting cells, there is also evidence of their role in impairing tumor progression.^[^ ^107^ ^]^ The diverse functions of CAFs within the TME are likely driven by distinct degrees of their activation at different stages of tumor progression.^[^ ^107^ ^]^ Therefore, selecting the most suitable marker for targeting CAFs is advisable. For instance, CD90+CD73+ peritumoral mesenchymal cells, which are considered to be precursors of fibroblasts, were recently demonstrated to suppress the immune system and to be responsible for ECM deposition in non‐squamous cell lung carcinoma.^[^ ^108^ ^]^ Therefore, they may be suitable markers to target tumor‐promoting CAFs.

Apart from CAFs, TAMs act as the main effectors of NP–cell interactions in the perivascular tumor environment.^[^ ^109^ ^]^ The innate tendency of TAMs to interact with NPs can be exploited to improve the accumulation of nanodrugs at the TME. For example, in the delivery of a cisplatin pro‐drug using polymeric NPs composed of poly(lactide‐*co*‐glycolide)‐*block*‐poly(ethylene glycol) methyl ether, TAMs acted as drug depot by internalizing most of the administered NPs and slowly releasing the active platin drug to adjacent cancer cells, thus hampering tumor growth.^[^ ^110^ ^]^ In another example, negatively charged gold NPs of 15, 55 and 100 nm (−9.6 ± 3, −7.8 ± 0.3, and −7.1 ± 0.2 mV, respectively) and silica NPs of 100 nm (slightly positively charged; 3.8 ± 0.2 mV) and 140 nm (slightly negatively charged; −0.7 ± 1.2 mV) were shown to mainly accumulate in TAMs at the tumor site, regardless of the presence of trastuzumab or folic acid as specific targeting ligands for cancer cells.^[^ ^111^ ^]^ The controlled targeting consistently reduced the amount of particles accumulating in clearing organs, although there was no significant difference in the percentage of particles reaching the cancer cells with or without targeting. Despite these limitations, targeting specific proteins expressed exclusively or predominantly by cancer cells, such as CD44 or epidermal growth factor receptor, has been explored as a strategy to deliver different cargos with the aim of suppressing fibrosis and inhibiting tumor growth; the cargos studied included collagenase, doxorubicin (DOX), and retinoic acid.^[^ ^112^ ^]^

As TAMs play a significant role in clearing NPs within the TME, employing immunotherapies to restrain their immune‐suppressive properties is promising. Similar to cancer cells and other immune‐suppressive myeloid‐derived suppressor cells, TAMs express immune checkpoint molecules, such as PD‐L1, so that leveraging their propensity for NP clearance can be strategically exploited to enhance anti‐tumor immunity. In this regard, the combination of ECM modulation with immunotherapies appears as a promising strategy to tackle cancer development and the immunosuppressive TME environment. For example, biocompatible dextran 20 kDa‐based NPs conjugated with hyaluronidases through a pH‐sensitive 3‐(bromomethyl)‐4‐methyl‐2,5‐furandione linker (NP size of ≈97 nm) were used as adjuvants in treatments performed with liposomes loaded with the photosensitizer chlorine e6 and immunotherapy with anti‐PD‐L1.^[^ ^113^ ^]^ Pretreatment with dextran‐based NPs conjugated with hyaluronidase led to an enhanced PDT efficacy and improved immune checkpoint blockade owing to the increased penetration of the liposomes and cytotoxic T lymphocytes (CD3+CD8+), following the enzymatic degradation of the tumoral ECM.^[^ ^113^ ^]^ In a different study, pH‐responsive clustered NPs of ∼100 nm in size and with a surface charge of ≈25 mV were used for the concomitant delivery of TGF‐β receptor inhibitor (LY2157299) and siRNA against PD‐L1; the NPs were obtained through self‐assembly of poly(ethylene glycol)‐*block*‐poly(ε‐caprolactone) polycaprolactone homopolymer and poly(amidoamine)‐*graft*‐polycaprolactone.^[^ ^114^ ^]^ The pH‐responsive NPs, which were sensitive to the acidic tumor environment, effectively reduced the deposition of collagen and promoted the infiltration of cytotoxic CD8+ T cells.^[^ ^114^ ^]^

Recent advances in immunotherapy have highlighted the possibility of reprogramming macrophages using a chimeric antigen receptor (CAR‐M), similar to chimeric antigen receptor T cells.^[^ ^115^ ^]^ The engineered CAR‐M was constituted of an scFv against a specific tumoral marker (e.g., HER2 or CD19), a transmembrane domain (e.g., CD8), and an intracellular domain for downstream signaling (e.g., CD3ζ). The engineered CAR‐M aimed at 1) enhancing phagocytosis of cancer cells, 2) stimulating the degradation of the ECM, and 3) improving antigen presentation and T cell activation.^[^ ^115^ ^]^ In summary, the study of TAMs as potential targets for nanomedicines seeks to either reprogram their activity toward immune‐stimulatory functions or utilize their phagocytic properties as drug reservoirs, as discussed in previous reviews.^[^ ^116^ ^]^

## 5 Future Perspectives

Since the first pioneering studies addressing the role of ECM in tissue homeostasis and disease development,^[^ ^117^ ^]^ it is now well established that ECM constitutes a dynamic environment undergoing continuous remodeling in response to mechanical and biochemical perturbations during tissue development or pathology progression, thus determining the stage of the disease.^[^ ^12^ ^]^

The distribution, abundance, and interaction of different spliced isoforms of ECM and ECM‐associated proteins, as well as their post‐translational modifications, are collectively referred to as matrisome, which determines the bio‐mechanophysical properties of specific tissues.^[^ ^118^ ^]^

Accordingly, capturing the diversity and complexity of the ECM and its interactions with NPs poses challenges that need to be addressed from multiple perspectives and requires concomitant investigation of materials properties, biological features, and clinical aspects.

### 5.1 In Vitro Models for Mimicking the ECM

To date, most studies on NP–cell interactions have been conducted on tissue culture surfaces such as glass or tissue culture plastic, which have rigidities in the gigapascals (GPa) range, exceeding the physiological stiffness of tissues by many folds.^[^ ^34^ ^]^ Additionally, under these conditions cells are often cultured on flat, rigid substrates, which fail to replicate the 3D complexity of the ECM present in living tissues. As a result, these models do not accurately capture the spatial and mechanical properties of the ECM, limiting their ability to reflect actual cellular behaviors and interactions with ECM components.

To overcome this limitation, ECM from different sources and a range of methodologies have been proposed to study NP–ECM interactions. The design of synthetic matrices, which are widely used in tissue bioengineering,^[^ ^119^ ^]^ aims to 1) support the implant of scaffolds with improved physiological properties for regenerative medicine or 2) study the pathophysiology of diseases. The biological properties of tissue‐specific ECMs also need to be carefully considered as the use of synthetic polymers may not always reproduce the functionality and physiology of biological matrices. For example, by using synthetic scaffolds, the susceptibility of ECM to spatiotemporal changes at the nanoscale level during the disease should be considered. These changes can determine the formation of stiff fibers without altering the overall bulk modulus of the fibrillar space, while inducing significant property modifications.^[^ ^42^ ^]^ Additionally, synthetic matrices may not act as a reserve of soluble factors or molecular signals that can influence the dynamic evolution of the TME during disease progression. For these reasons, it is advisable to use scaffolds that consider the interplay between soluble factors and ECM remodeling.^[^ ^120^ ^]^ For example, dynamic molecular strategies have been employed to create ECM‐mimicking surfaces that can present or remove ligands in response to specific stimuli, such as light, pH, or electric field.^[^ ^121^ ^]^ By using reversible covalent and noncovalent bonds, these materials allow precise and reversible control over cellular interactions, supporting applications in tissue engineering and regenerative medicine.^[^ ^121^ ^]^ This approach allows biomaterials to interact adaptively with cells, promoting desired cellular behaviors and more accurately recapitulating the biological and physicochemical properties of the TME. This, in turn, may help to improve the assessment of biomaterials in preclinical setups.

Another promising approach to overcome the limitations of synthetic matrices is the application of 3D‐bioprinted models, where a variety of cells—from cell culture lines to patient‐derived cells—can be embedded in a hydrogel scaffold that aims to resemble the pathophysiology of tumors and is being used to predict therapeutic outcomes and design personalized therapies.^[^ ^122^ ^]^ These matrices can be composed of different natural ECM components, such as fibrin, laminin, hyaluronic acid, heparan sulfate, and gelatin (a hydrolyzed form of collagen), and have the advantage of being biocompatible and tunable by the remodeling activity of cancer cells.^[^ ^123^ ^]^ In addition, 3D‐bioprinted scaffolds can be implemented with a vasculature network for further recapitulating the characteristic of the TME and studying angiogenesis and cell invasion processes during disease development.^[^ ^124^ ^]^ 3D‐Bioprinted models can also provide better outcomes in terms of drug screening and disease progression studies than Matrigel.^[^ ^125^ ^]^ The latter is a widely used solution of ECM components derived from murine Engelbreth–Holm–Swarm sarcoma and is extensively used for the generation of in vitro 3D tumor models and evaluation of NP–ECM interactions.

Ideally, the most representative model for studying NP–ECM interactions relies on the use of patient‐derived samples. The ECMs from patient tumors and tissues have been used in vitro to better recapitulate the functions and roles of ECM composition and structure in the development of diseases or to design scaffolds for regenerative medicine.^[^ ^126^ ^]^ Efficient and conservative decellularization protocols^[^ ^127^ ^]^ and techniques, such as high‐resolution imaging,^[^ ^128^ ^]^ quantitative proteomics,^[^ ^129^ ^]^ and second harmonic generation,^[^ ^130^ ^]^ have led to the disclosure of important aspects of ECM biology.^[^ ^58^ ^]^ These findings can be useful at the preclinical stage for developing personalized nanomedical approaches based on the ECM features of a given tumor. Nevertheless, accessibility to these materials is limited to collaborations with hospitals, the presence of suitable equipment, and the management of ethics documentation.^[^ ^131^ ^]^ An ideal platform to evaluate mechanotherapeutics and the efficiency of NP‐mediated drug delivery in general, starting from patients' tissues, entails the use of cancer organoids (or tumoroids). These can recapitulate the peculiar cellular and acellular composition of the TME, its architecture, and its functionality.^[^ ^132^ ^]^ These models offer great promise in the field of personalized therapy and can also be used to assess the efficiency of drugs against aberrant ECM deposition and remodeling in a variety of tumors.^[^ ^133^ ^]^ However, a limitation is that organoids may not fully replicate the ECM characteristics found in the original tissue. This is because organoids are typically derived from digested and processed samples, which can alter the composition and structure of the ECM. This aspect requires careful consideration when interpreting results from organoid cultures. The outcomes of these studies may need to be complemented with other model systems, such as scaffolds originating from decellularized orthotopic tissues,^[^ ^126a^ ^]^ or with in vivo studies to comprehensively understand the complexities of tissue biology and drug responses.

### 5.2 Designing NP Properties for Interactions with the ECM

The size, shape, and surface charge of NPs have been highlighted as key factors that determine their tumor penetration efficiency, as discussed sections *Physicochemical Properties of the ECM and NPs* (Section 4.1) and *ECM Density* (Section 4.2). In terms of shape, anisotropic particles, such as nanorods, have been shown to penetrate ECM better than spherical particles. Particle elasticity is also likely to play a prominent role in promoting ECM penetration. Softer NPs have been shown to accumulate significantly more in tumors than their stiffer counterparts.^[^ ^134^ ^]^ Although this is likely to be a direct consequence of the different interactions that NPs display with different cell types,^[^ ^135^ ^]^ it is reasonable to assume that soft NPs can extravasate and penetrate the ECM more efficiently than stiffer NPs.

Regarding surface charge, neutral and anionic NPs exhibit better ECM penetrability than cationic NPs, mainly due to reduced interactions with the charged components of the ECM, in particular the negatively charged hyaluronan and chondroitin sulfate. Therefore, considerations on this matter need to account for protein corona, colloidal stability issues, and target tissue.^[^ ^136^ ^]^

In terms of size, NPs smaller than 100 nm can diffuse through the ECM more efficiently than larger NPs. However, to avoid renal clearance, NPs with a threshold size of 10 nm are preferable; they may also display longer bioavailability and biodistribution.^[^ ^137^ ^]^ Considering the latter two properties, hydrophilicity is an important feature for the circulation of NPs in the body and has been demonstrated to improve the penetration of NPs in the ECM. The use of PEG or other amphiphilic ligands is essential for the efficient penetration of NPs in the ECM. In particular, the grafting density of these ligands correlates with increased diffusion of nanomedicines in the TME, likely due to reduced interactions with peripheral phagocytic cells and increased diffusion in the ECM network.^[^ ^138^ ^]^

Regarding composition, a wide range of materials have been used for the synthesis of NPs, with polymer‐based, metal, and lipid‐based particles being the most representative. Although the choice of material depends on the intended application, affordability, long‐term biocompatibility, and scalability are key parameters for their successful clinical translation. For instance, polymeric particles are ideal for drug delivery, metal particles for physical treatments such as MHT and PTT, and LNPs for nucleic acid therapy. Stimuli‐responsive polymers can be both advantageous and disadvantageous. Smart nanosystems that can respond to the changes of the ECM (such as MMP‐sensitive materials) and the TME (such as pH‐sensitive materials) are appealing. However, in most cases, they rely on complex synthetic polymers that are poorly biocompatible and costly, display low yields and poor scalability, and require lengthy preparation.^[^ ^139^ ^]^ Alternatively, biocompatible and biodegradable polysaccharides, such as neutral glycogen or anionic alginate that are naturally abundant and display tunable properties, may be used as coating materials or as stand‐alone NPs for various drug delivery applications.^[^ ^140^ ^]^

Metal NPs, such as iron and gold NPs, have been extensively used for MHT and PTT applications.^[^ ^141^, ^142^ ^]^ Provided that metal NPs can accumulate at tumor sites at a sufficiently high concentration for an effective treatment—not only via intratumoral injection but more importantly via intravenous injection—their use may represent an efficient strategy to undermine the ECM structure and organization, thereby allowing the penetration of drugs or other nanomedicines as a second line of treatment.

Lipid‐based NPs have emerged as the main delivery vectors in nanomedicine for a range of compounds, from drugs to proteins and nucleic acids, in virtue of their unique properties in terms of composition, flexibility, and scalability.^[^ ^41^ ^]^ Varying the composition and nature of the lipids allows for selective organ targeting (SORT) and increased likelihood of accumulation at the desired site of the body.^[^ ^143^ ^]^ The dependence of protein corona composition on the lipid composition of LNPs, and its relationship with SORT, has been established.^[^ ^144^ ^]^ In contrast, the role of the ECM composition in different tissues, particularly tumoral ECM, on the fate of LNPs composed of different formulations has been poorly investigated. Understanding this role could uncover important aspects of the SORT mechanism and facilitate the development of NPs with improved targeting specificity.

Along with the structural identity of the NPs, the presence of targeting ligands grafted onto the NP surface is likely to influence the interactions with ECM components. In contrast to the effect of protein corona on the surface properties and biological identity of NPs that has been extensively studied,^[^ ^145^ ^]^ the role of ECM components has been less investigated. In this context, targeted interstitial transport may represent a promising strategy by exploiting the presence of selective molecular markers in the tumor ECM to facilitate the accumulation and release of drugs. Regarding targeting, although the delivery of drugs loaded into NPs can be more influenced by the hindrance of the ECM than a free‐drug formulation, it is important to highlight that as nanodrugs can load and release several drug molecules within the same moiety, the therapeutic efficacy of NPs is likely to remain competitive, if not superior.^[^ ^146^ ^]^

**Figure 12:**
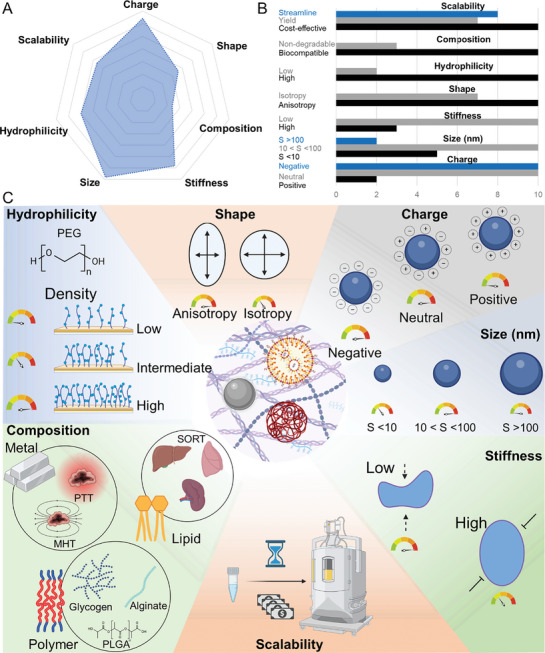
Factors to consider in the design of NPs for cancer treatment to overcome or exploit the properties of the ECM and TME. A) Arbitrary radar graph representing the balance among the different properties that NPs should ideally exhibit for optimal interaction with the ECM. The graph illustrates the relative importance of each selected property based on arbitrary values. B) Bar plot showing the importance of the NP properties selected from the radar graph in (A). On a scale of values from 0 to 10, 10 is the most important and 0 is the least important for interaction with the ECM. C) Schematic showing the different nanomaterial properties that inform the rational design of NPs for their interaction with the ECM to maximize drug delivery and efficacy in cancer treatment. In addition to physicochemical properties—size, shape, charge, composition, hydrophilicity, and stiffness—scalability factors such as low cost, high yield, and time efficiency should be considered in the design of NPs.

In summary, negatively charged anisotropic soft NPs that are smaller than 100 nm seem to represent the most suitable choice to maximize the penetration of nanodrugs in the ECM. While also considering the affordability and scalability of the nanomaterials, which ultimately would dictate their successful clinical translation, it is possible to weigh the different properties of the NPs (as discussed in Section 3) according to their relevance in overcoming the ECM barrier at the tumor (**Figure** **12**). For instance, properties such as charge, size, and surface functionalization are more critical for optimizing cellular uptake and interaction with the ECM, while scalability and composition become key factors for large‐scale manufacturing and regulatory approval.

However, a consensus for the rational design of NPs is challenged by the intrinsic complexity of the ECM and is far from a “one size fits all” approach. Moreover, this design cannot overlook other biological barriers that nanomedicines face in the human body, such as the protein corona, clearance by the reticuloendothelial system, and interaction with the MPS.^[^ ^147^ ^]^ Notwithstanding these key factors, the implementation of ECM models at the preclinical level, which accurately represent its complexity,^[^ ^148^ ^]^ may greatly improve the initial screening of nanodrugs and help in the selection of optimal physicochemical parameters, considering the nature of a given tumor on a case‐by‐case basis.

### 5.3 Mechanotherapy in Cancer Treatment

Different therapeutic approaches have been developed with the aim of increasing the permeability of tumors.^[^ ^78a^ ^]^ Anti‐fibrotic therapies, focused on the inhibition of the TGF‐β pathway, have been applied for reducing ECM deposition and hamper cancer cell proliferation and metastasis.^[^ ^114^, ^149^ ^]^ Although the disruption of the tumor ECM allows easier interstitial transport of therapeutics, it also lowers the barriers for tumor cells to undergo metastasis, thus questioning the clinical relevance of treatments aimed at impairing ECM deposition.^[^ ^150^ ^]^ Hence, to circumvent this drawback, a different approach using metalloproteinase inhibitors (MPIs) to halt tumoral ECM remodeling and limit cancer cells motility, extravasation, and metastasis has been attempted.^[^ ^151^ ^]^ However, the lack of specificity for cancer‐associated MMPs could affect the migration of effector immune cells at the tumor site, reduce the activation of cytokines such as TNF‐α, which play roles in promoting inflammation, anti‐tumor immunity and impair tissue repair.^[^ ^151^ ^]^ Though MPIs offer a strategy for reducing metastasis and angiogenesis, their broad impact on immune function and a lack of specificity pose challenges, particularly in balancing immune activity and tumor suppression.

Even though an increased stiffness is generally regarded as hallmark of cancer, only a few studies have demonstrated that a reduced and soft ECM can promote tumor invasion and immune suppression.^[^ ^152^ ^]^ For instance, although the Hippo pathway kinases LATS1/2 are traditionally regarded as tumor suppressors (as discussed in *ECM Mechanobiology*, Section 3), their inhibition in murine melanoma, head and neck squamous cell carcinoma and breast cancer models has been shown to enhance tumor immunogenicity.^[^ ^152b^ ^]^ This occurs through the release of nucleic‐acid‐rich extracellular vesicles, which activate immune pathways, including the Toll‐like receptors and type I interferon pathways, thereby stimulating anti‐tumor immune responses.^[^ ^152b^ ^]^ Targeting LATS1/2 in specific tumoral environments could, therefore, represent a promising strategy to increase the immunogenicity of tumors and improve outcomes in cancer immunotherapy by enhancing immune detection of cancer cells. In another example, ovarian cancer cells have been shown to preferentially adhere to softer microenvironments, where they exhibit an increasingly malignant phenotype.^[^ ^152a^ ^]^ In soft conditions, the activation of the Rho–ROCK pathway enhances cell malignancy by increasing ovarian cancer cell adhesion, proliferation, migration, and chemoresistance. This mechanotransduction effect on soft matrices, rather than stiffer ones, induces a more mesenchymal, invasive, cell phenotype, facilitating metastatic spread and resistance to carboplatin treatment.^[^ ^152a^ ^]^

Notwithstanding these limitations, mechanotherapy has recently emerged as a promising approach to normalize the ECM by improving tumor perfusion and reducing hypoxia and IFP, as our understanding of the relationship between mechanobiology and cancer development continues to evolve.^[^ ^15^ ^]^ Several treatments targeting mechanotransduction pathways involved in different pathologies, such as integrin, angiotensin, FAK, and TGF‐β, have shown promising outcomes at the preclinical stage and are undergoing clinical trials (**Table** **2**).^[^ ^15^ ^]^ Besides the use of LOS for targeting angiotensin receptor, defactinib, a specific FAK inhibitor, efficiently blocks the Akt/PKB signaling pathway and reduces the expression of several oncogenes involved in cell survival, motility, and proliferation.^[^ ^153^ ^]^ Another example is M7824, a bispecific fusion protein that is designed to concomitantly bind the TGF‐β receptor and PD‐L1, thus simultaneously blocking the fibrotic cascade triggered by TGF‐β pathway and the immune checkpoint blockade that represses the activation of cytotoxic T cell.^[^ ^154^ ^]^ Conversely, the application of integrin inhibitors has been more challenging to date. Despite the extensive number of small molecules and antibodies developed in the last decades, the use of integrin inhibitors has yet to produce successful clinical outcomes.^[^ ^155^ ^]^ Although their use has shown promising preclinical stage results, further progress is required at the clinical stage, which is limited due to the lack of specificity, pathway redundancy, and inter‐patient variability of integrin receptors.^[^ ^155^ ^]^

**Table 2:** Selection of mechanotherapeutic drugs in a clinical trial for the treatment of cancer.

Identifier^a)^	Condition	Drug	Target	Combination therapy	Status
NCT01008475	Metastatic colorectal cancer	Abituzumab	**αν integrins**	+ Cetuximab + Irinotecan	Completed (no benefit)
NCT00848510	Colorectal and ovarian cancer with liver metastasis			Monotherapy	Completed (no benefit)
NCT03688230	Metastatic colorectal cancer			+ Cetuximab + FOLFIRI	Withdrawn
NCT05669482	Metastatic pancreatic cancer	Defactinib	**FAK**	+Avutometinib + Gemcitabine + Nab‐paclitaxel	Phase I
NCT02546531	Advanced pancreatic cancer			+ Pembrolizumab + Gemcitabine	Phase 1
NCT05512208	Refractory gynecological cancer			+ Avutometinib	Phase 2
NCT04620330	NSCLC				Phase 2
NCT05787561	Advanced or recurrent mesonephric gynecologic Cancer				Phase 2
NCT03727880	Pancreatic ductal adenocarcinoma			+ Pembrolizumab	Phase 2
NCT02523014	Meningiomas	GSK2256098		+ Abemaciclib + Capivasertib +Vismodegib	Phase 2
NCT03941093	Pancreatic cancer	Pamrevlumab	**CTGF**	+ Gemcitabine + Nab‐paclitaxel or FOLFIRINOX	Phase 3
NCT04229004	Metastatic pancreatic cancer				Phase 3
NCT04729725	Solid tumor	SAR439459	**TGF‐β**	+ Cemiplimab	Phase 1
NCT04031872	Colorectal cancer	LY3200882		+ Capecitabine	Phase 1 Phase 2
NCT05322408	Pancreatic cancer	HCW9218 – Bifunctional TGF‐β antagonist/IL‐15 protein complex		Monotherapy	Phase 2
NCT05198505	Advanced tumors	TQB2868 ‐ Bifunctional TGF‐β antagonist/PD‐1 protein complex		Monotherapy	Phase 1
NCT05998941	Metastatic cervical cancer			+ Paclitaxel + Cisplatin + Bevacizumab	Phase 2
NCT05653284	Advanced tumors	AK130 – TIGIT/TGF‐β bifunctional fusion protein		Monotherapy	Phase 1
NCT04976218	EGFR‐positive solid tumors	CAR‐EGFR‐TGFβR‐KO T cells		CAR T cells KO for TGF‐β receptor II and specific for EGFR	Phase 1
NCT04064190	Urothelial carcinoma failing checkpoint inhibition	Vactosertib		+ Durvalumab	Phase 2
NCT05145569	Metastatic ovarian cancer	Bintrafusp alfa Bifunctional TGF‐β antagonist/PD‐L1 protein complex		+ Carboplatin + Paclitaxel	Phase 1
NCT05005429	Advanced pleural mesothelioma			Monotherapy	Phase 2
NCT05077800	Pancreatic cancer	Losartan	**AT1R**	+ FOLFIRINOX + GSK3B inhibitor	Phase 2
NCT03900793	Osteosarcoma			+ Sunitinib	Phase I
NCT05861336	Pancreatic cancer			+ Gemcitabine + Nab‐paclitaxel	Phase 2
NCT03563248				+ Nivolumab +FOLFIRINOX	Phase 2
NCT05097248	TNBC			+ Camrelizumab + Liposomal DOX	Phase 2

^a)^ Sourced from Clinicaltrials.gov. **Cetuximab**: anti‐EGFR antibody; **Irinotecan**: DNA topoisomerase inhibitor; **FOLFIRI**: therapeutic regimen of leucovorin calcium (folinic acid), fluorouracil, irinotecan hydrochloride; **Avutometinib**: inhibitor of Ras‐Raf‐MEK‐ERK pathway; **Gemcitabine**: chemotherapeutic antimetabolite, interrupts DNA synthesis; **Nab‐paclitaxel**: albumin‐bound paclitaxel, causes mitotic arrest by stabilizing microtubules; **Pembrolizumab**: anti‐programmed death‐ligand 1 (anti‐PD‐L1) antibody; **NSCLC**: non‐small cell lung cancer; **FAK**: focal adhesion kinase; **Abemaciclib**: cyclin‐dependent kinase (CDK) inhibitor selective for CDK4 and CDK6; **Capivasertib**: Akt kinase inhibitor; **Vismodegib**: Hedgehog pathway inhibitor; **CTGF**: connective tissue growth factor; **FOLFIRINOX**: therapeutic regimen of leucovorin calcium (folinic acid), fluorouracil, irinotecan hydrochloride, and oxaliplatin; **TGF‐β**: transforming growth factor β; **Cemiplimab**: anti‐PD‐L1 antibody; **Capecitabine**: chemotherapeutic antimetabolite, interrupts DNA synthesis; **Bevacizumab**: anti‐vascular endothelial growth factor (VEGF) antibody; **TIGIT**: T cell immune receptor with immunoglobulin (Ig) and immunoreceptor tyrosine‐based inhibitory motif (ITIM) domains, inhibitor of anti‐tumor responses; **EGFR**: epidermal growth factor receptor; **CAR T cells**: chimeric antigen receptor T cell; **Durvalumab**: anti‐PD‐L1 antibody; **AT1R**: angiotensin receptor II; **GSK3B**: glycogen synthase kinase‐3 β; **Sunitinib**: multitargeted receptor tyrosine kinase inhibitor; **Nivolumab**: anti‐PD‐L1 antibody; **TNBC**: triple‐negative breast cancer; **Camrelizumab**: anti‐programmed cell death protein 1 (anti‐PD1) inhibitor.

### 5.4 Mechanotherapy and Nanomedicine: The Road to a Synergistic Approach

**Figure 13:**
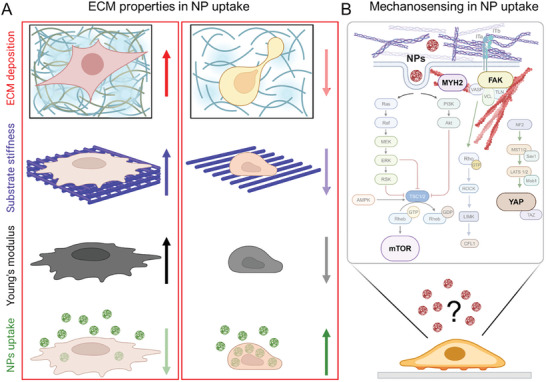
Mechanical properties of the ECM and the mechanosensing components of the cells contribute to cell–NP interactions. A) The abundant deposition of the ECM at the tumor influences the mechanical properties of the TME, driving the mechanical state of the cells to increased stiffness and ultimately reducing the internalization of NPs by cancer cells. Targeting ECM deposition in cancer may effectively enhance tumor penetration of nanomedicines, concurrently reduce the membrane tension of cancer cells, and thereby increase NP endocytosis. B) Different mechanosensing proteins, such as mTOR, YAP, myosin II (MYH2), and FAK, have been proposed to regulate cell–NP interactions through a mechanobiology cascade. However, the precise role of each protein, their interplay, and the involvement of other components in this process remain unclear. The understanding of this phenomenon is still in its infancy.

The application of combined treatments using mechanotherapy, immunotherapy, and chemotherapy is steadily increasing (Table 2).^[^ ^156^ ^]^ These therapies can be combined with nanomedicine to improve the delivery of NPs to tumors while suppressing the pro‐tumorigenic stimuli of abnormal ECM, thereby addressing the disease from multiple facets.^[^ ^157^ ^]^ Considering the combination of mechanotherapy and nanomedicine, we have recently shown that YAP activity hinders NP binding and internalization by cancer cells.^[^ ^25b^ ^]^ This phenomenon has been ascribed to several functions that YAP exerts in cancer cells, including the regulation of cell mechanosensing, the transcriptional inhibition of proteins involved in endocytosis, the perturbation of cell membrane tension and organization, and the promotion of the deposition of an abundant and rich ECM network.^[^ ^25b^ ^]^ Few studies have highlighted the role of the mechanical properties of substrates and cell mechanobiology in cell–NP interactions (**Figure** **13**A).^[^ ^158^ ^]^ Recently, Voigt et al. demonstrated that the uptake of carboxylated polystyrene NPs of 40 and 200 nm in size by HeLa cervical cancer cells increased on soft substrates with stiffness of <5 kPa, on which cells exhibited lower cell adhesion, membrane stiffness, and surface area compared with cells grown on stiffer substrates.^[^ ^159^ ^]^ Lee et al. reported that the reduction of focal adhesion, membrane stiffness, and cell spreading exhibited by A549 lung adenocarcinoma cells and J774A.1 murine macrophage cell line correlated with a decrease in substrate stiffness, leading to increased uptake of 120 and 360 nm silica NPs.^[^ ^160^ ^]^ Moreover, cells grown on soft substrates displayed a higher YAP expression than cells cultured on standard tissue culture plates (which have Young's modulus in the GPa range) and protein expression correlated with increased NP uptake. Although these findings contrast with previous observations,^[^ ^25^, ^161^ ^]^ it is worth considering that the different mechanosensing proteins could have differing roles depending on the state of the cells, their differentiation, and the nature of the cell–substrate interaction, which consequently influence cell–NP interaction mechanisms. In another example, Athirasala et al. demonstrated a higher transfection efficiency of LNP‐mRNA in human bone marrow‐derived mesenchymal stem cells for soft substrates (<10 kPa) compared with that for stiffer substrates (>40 kPa), as well as greater relative mRNA translation as a result of higher internalization of LNPs by the cells.^[^ ^162^ ^]^ The findings of these studies suggest the effects of different regulators in determining cell–NP interaction mechanisms. The roles of FAK, YAP, myosin II, or mTOR on NP uptake,^[^ ^25^, ^159^, ^160^, ^162^ ^]^ and their interplay and the possible involvement of other mechanosensing machineries in the process remain unclear (Figure 13B).

Yankaskas et al. have recently shown that cancer cell migration and extravasation (and thus the ability to enter vascular vessels and form distant metastasis) depend on the response of cells to shear stress and the activation of RhoA GTPase.^[^ ^37^ ^]^ In particular, the inhibition of Rho‐associated protein kinase (ROCK) rendered the cells unresponsive to shear stress and promoted cell extravasation. As ROCK activity positively correlated to that of YAP, ERM proteins, and membrane tension, the study reveals that migrating cells may be more sensitive to NP targeting owing to changes in membrane forces and organization, as previously hypothesized by Wei et al.^[^ ^158b^ ^]^ This evidence suggest the possibility of exploiting the deregulation of mechanosensing pathways in cancer cells to selectively target highly migrating cells and cancers with pro‐metastatic features. Cell mechanics are also involved in the organization and structural assembly of the plasma membrane and consequently may significantly influence the kinetics of cell–NP association.^[^ ^163^ ^]^

Further studies are needed to clarify the interconnection between mechanosensing and nanomaterials and particularly to assess its pre‐clinical and clinical value. Ideally, the role that cell mechanobiology plays in cell–NP interactions and the functions of the main pathways and effectors may have in improving the delivery of NPs to cancer cells could be exploited to improve the design of NPs, with the development and characterization of more efficient nanomaterials in terms of interactions with cell membranes.

In this matter, NPs engage with membranes via hydrophobic and electrostatic interactions, which are influenced by the characteristics of the NPs and the curvature of the membranes.^[^ ^147^ ^]^ The receptor‐ligand approach is widely used to enhance these interactions by modifying the surfaces of nanomedicines. Various nanomedicines have been developed to target different membranes for better therapeutic outcomes,^[^ ^164^ ^]^ and it has been shown that physically adsorbed ligands tend to aggregate within lipid bilayers, influencing membrane integrity and boosting cellular uptake.^[^ ^147^ ^]^ Therefore, the choice of the right ligand and the most appropriate bioconjugation strategy is essential for achieving effective targeting.^[^ ^147^ ^]^

It is worth mentioning that mechanical forces can be strategically harnessed in an active way to overcome the challenges posed by the tumoral ECM, aiming to modulate immune responses and enhance drug delivery. To this end, medical nanorobots have been designed to exploit power sources such as near‐infrared light, ultrasound, or magnetic forces.^[^ ^165^ ^]^ These nanorobots differ from conventional nanomaterials as they rely on active power systems to target the desired cell or tissue rather than passive mechanisms.^[^ ^165^ ^]^ For instance, magnetic nanorobots can generate mechanical cues that guide macrophage polarization, allowing it to shift immune responses to a more therapeutic state.^[^ ^166^ ^]^ Additionally, biomimetic nanorobots with near‐infrared‐triggered capabilities can penetrate dense tumor stroma, breaking down ECM barriers and improving drug distribution within tumors.^[^ ^167^ ^]^ These innovative approaches leverage mechanical forces to overcome ECM‐related obstacles, opening new possibilities for effective cancer therapies targeting the TME.

## 6 Conclusion

A comprehensive understanding of the relationship between the evolving ECM and tumor cells is essential for advancing effective cancer screening and prevention programs. This knowledge would enable the prediction of disease progression and the identification of functional and compositional changes occurring within the TME during disease progression.^[^ ^1^ ^]^ Similarly, the development of advanced nanodrugs should consider the properties of the tumor ECM that determine their successful application in cancer treatment. As cell mechanobiology is intrinsically connected to the nature of the ECM, a thorough understanding of the mechanosensing pathways involved in bio–nano interactions can also be leveraged to improve the design of nanomaterials for cancer treatment. Based on these assumptions, research on new targets and drugs with modulated functions may, in the future, pave the way to the development of effective next‐generation nanotherapies to address the challenges posed by cell targeting and selective drug delivery through nanomedicines. Finally, the stratification of patients based on disease‐specific ECM features and mechanobiology signatures, such as key components associated with poor prognosis and evidence of stromal invasion, is essential for tailoring the most appropriate therapeutic regimen, leveraging the diverse array of anticancer treatments available, including nanomedicines, to achieve optimal treatment outcomes.

## Conflict of Interest

The authors declare no conflict of interest.
